# Synthesis and Antibacterial Evaluation of a New Series of *N*-Alkyl-2-alkynyl/(*E*)-alkenyl-4-(1*H*)-quinolones

**DOI:** 10.3390/molecules17078217

**Published:** 2012-07-09

**Authors:** Abraham Wube, Juan-David Guzman, Antje Hüfner, Christina Hochfellner, Martina Blunder, Rudolf Bauer, Simon Gibbons, Sanjib Bhakta, Franz Bucar

**Affiliations:** 1 Department of Pharmacognosy, Institute of Pharmaceutical Sciences, Karl-Franzens-University Graz, Universitätsplatz 4, A-8010 Graz, Austria; Email: abraham.wube@uni-graz.at (A.W.); christina.hochfellner@uni-graz.at (C.H.); martina.blunder@uni-graz.at (M.B.); rudolf.bauer@uni-graz.at (R.B.); 2 Department of Biological Sciences, Institute of Structural and Molecular Biology, Birkbeck, University of London, Malet Street, London WC1E 7HX, UK; Email: talauma@gmail.com (J.-D.G.); s.bhakta@bbk.ac.uk (S.B.); 3 Department of Pharmaceutical and Biological Chemistry, UCL School of Pharmacy, 29-39 Brunswick Square, London WC1N 1AX, UK; Email: simon.gibbons@ucl.ac.uk; 4 Department of Pharmaceutical Chemistry, Institute of Pharmaceutical Sciences, Karl-Franzens-University Graz, Universitätsplatz 1, A-8010 Graz, Austria; Email: antje.huefner@uni-graz.at

**Keywords:** *N*-alkyl-2-alkynyl/(*E*)-alkenyl-4(1*H*)-quinolone, antimycobacterial, MRSA, cytotoxicity

## Abstract

To gain further insight into the structural requirements of the aliphatic group at position 2 for their antimycobacterial activity, some *N*-alkyl-4-(1*H*)-quinolones bearing position 2 alkynyls with various chain length and triple bond positions were prepared and tested for *in vitro* antibacterial activity against rapidly-growing strains of mycobacteria, the vaccine strain *Mycobacterium bovis* BCG, and methicillin-resistant *Staphylococcus aureus* strains, EMRSA-15 and -16. The compounds were also evaluated for inhibition of ATP-dependent MurE ligase of *Mycobacterium tuberculosis*. The lowest MIC value of 0.5 mg/L (1.2–1.5 µM) was found against *M. fortuitum* and *M. smegmatis*. These compounds displayed no or only weak toxicity to the human lung fibroblast cell line MRC-5 at 100 µM concentration. The quinolone derivatives exhibited pronounced activity against the epidemic MRSA strains (EMRSA-15 and -16) with MIC values of 2–128 mg/L (5.3–364.7 µM), and *M. bovis* BCG with an MIC value of 25 mg/L (66.0–77.4 µM). In addition, the compounds inhibited the MurE ligase of *M. tuberculosis* with moderate to weak activity showing IC_50_ values of 200–774 µM. The increased selectivity towards mycobacterial bacilli with reference to MRC-5 cells observed for 2-alkynyl quinolones compared to their corresponding 2-alkenyl analogues serves to highlight the mycobacterial specific effect of the triple bond. Exploration of a terminal bromine atom at the side chain of *N*-alkyl-2-(*E*)-alkenyl-4-(1*H*)-quinolones showed improved antimycobacterial activity whereas a cyclopropyl residue at N-1 was suggested to be detrimental to antibacterial activity.

## 1. Introduction

Since the discovery of the bacterial ethiology of tuberculosis (TB) by Robert Koch in 1882, various attempts have been devised to treat infections caused by *Mycobacterium tuberculosis*. Streptomycin and *p*-aminosalicylic acid were the first drugs introduced to treat TB, followed by isoniazid, pyrazinamide, rifampicin and ethambutol, which were championed as the first-line agents [1,2]. However, the emergence of strains that are resistant to these weapons necessitated further drug options that led to the second- and third-generation antibiotics. In the early 1990’s fluoroquinolones were introduced to tackle the ever increasing danger of bacterial resistance and have enjoyed great success against both Gram-positive and Gram-negative bacteria and certain anaerobes. Unfortunately, mutations resulting from spontaneous chromosomal alterations, and their widespread overuse and misuse have contributed significantly to quinolone resistance [3]. Currently there is increasing concern regarding the paucity of new antimycobacterial therapeutic agents that are coming onto the market in spite of increased mycobacterial resistance. Therefore, new drugs with new modes of action are required in view of worldwide-emerging multi drug resistant (MDR-) and extensively drug resistant (XDR) *M. tuberculosis* strains [4].

Cell wall biosynthesis is one of the prime targets for drug discovery, because it is an essential process in the bacterial life cycle. Enzymes involved in the later stage of bacterial cell wall biosynthesis have been the targets of successfully marketed antibacterial drugs such as β-lactams including pencillins, carbapenems and cephalosporins, and vancomycin as well as several promising agents in advanced clinical study such as telavancin and capuramycin [5]. In contrast, except for fosfomycin no successful antibacterial agents targeting the first cytoplasmic steps of murein biosynthesis have appeared on the market.

Mur ligases are enzymes that catalyze in a stepwise fashion the early cytoplasmic reactions of bacterial cell wall biosynthesis, and one of these are the ATP-dependent amide forming ligases of *M. tuberculosis*, being MurE the enzyme responsible for catalyzing the addition of *meso*-diaminopimelic acid (*m*-DAP) to the nucleotide precursor UDP-MurNAc-L-Ala-D-Glu to form UDP-MurNAc-L-Ala-D-Glu-*m*-DAP [6]. We have recently disclosed *N*-methyl-4(1*H*)-quinolones bearing an alkenyl moiety at position 2 as a novel class of *M. tuberculosis* MurE inhibitors [7]. The compounds inhibited *M. tuberculosis* MurE *in vitro* in the micromolar range, making them successful anti-TB scaffolds for further rounds of medicinal chemistry improvement.

Our investigations into the design and synthesis of this class of antimycobacterials started with the initial discovery of the potent antimycobacterial properties of quinolone alkaloids isolated from the Chinese medicinal plant *Euodia rutaecarpa* [8,9]. In recent reports, we have described the synthesis, antimycobacterial and cytotoxicity evaluation of a series of *N*-methyl-2-alkenyl-4(1*H*)-quinolones [10,11]. We observed that quinolones having alkenyl moieties at C-2 displayed superior inhibitory effect compared to their alkyl analogues and subsequent mechanistic investigations indicated that the antimycobacterial activity of these compounds is related to inhibition of *M. tuberculosis* MurE ligase [7]. We are particularly interested in the synthesis and biological evaluation of other *N*-alkyl-4(1*H*)-quinolones, since the antimycobacterial mechanism of action is assumed to be different from the currently used anti-TB agents, and also different from DNA gyrase/topoisomerase inhibition of clinically-used fluoroquinolones that have an essential free carboxylic group at position C-3 [12], absent in active *N*-alkyl-4(1*H*)-quinolones. Based on these findings we decided to continue our investigation on modification of the aliphatic side chain at position 2, and the present study was principally undertaken to explore the impact of the triple bond on the antibacterial and cytotoxic activities of the quinolones at C-2. Here we report the synthesis and biological evaluation of *N*-alkyl-4(1*H*)-quinolones having alkynyls at C-2 with the intent of elucidating the role that these groups may play in the *in vitro* antibacterial profile against fast and slow growing strains of mycobacteria, and methicillin-resistant *S. aureus* strains keeping in mind potential correlations with the *M. tuberculosis* MurE enzyme inhibitory data. In addition, the influence of a terminal bromine atom at the side chain on the antimycobacterial activity was evaluated. The cytotoxicity profile of the synthetic derivatives was assessed against the human lung fibroblast cell line MRC-5.

## 2. Results and Discussion

### 2.1. Synthesis

**Scheme 1:**
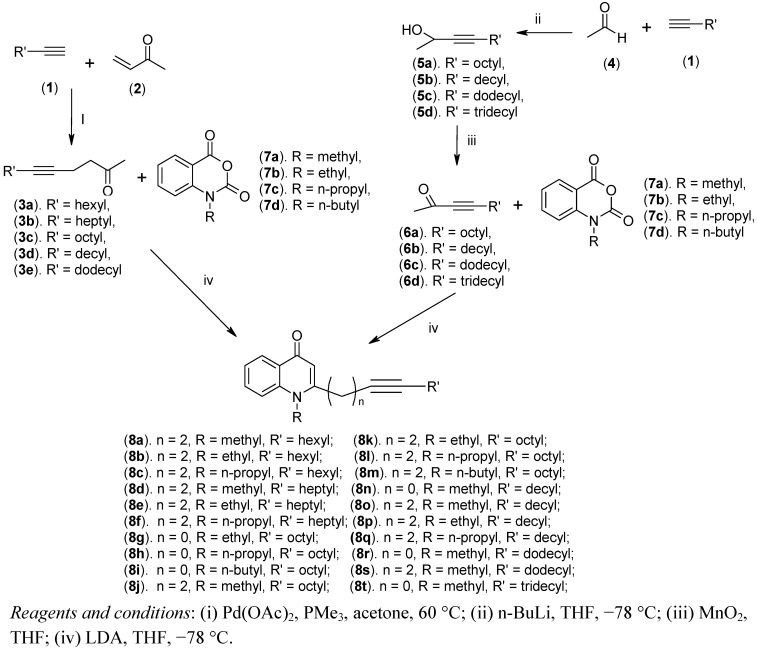
Synthesis of *N*-alkyl-2-alkynyl-4(1*H*)-quinolones.

As shown in Scheme 1, our rational design for the synthesis of *N*-alkyl-2-alkynyl-4(1*H*)-quinolones was based on the replacement of the double bond with a triple bond, while maintaining the basic quinolone skeleton. The synthetic route targeting compounds **8a–t** is given in Scheme 1. The key intermediates, **3a–e** and **6a–d**, which are essential for the synthesis of our target quinolone derivatives were obtained in two ways. Pd(OAc)_2_ and PMe_3_ mediated direct coupling of methyl vinyl ketone (**2**) and 1-alkynes **1** in acetone afforded 5-alkynyl-2-ones **3a–e** [13]. The 3-alkynyl-2-ones **6a–d**, on the other hand, were prepared according to the Xing and O’Doherty procedure [14] by treatment of the terminal alkynes **1** with acetaldehyde in the presence of *n*-BuLi in THF and subsequent oxidation with MnO_2_. The synthesis of this series of 4(1*H*)-quinolones was accomplished by the well-known condensation of methyl alkynyl ketones **3a–e** and **6a–d** with *N*-alkyl isatoic acid anhydrides **7a–d** in presence of LDA in THF at −78 °C. 

**Scheme 2:**
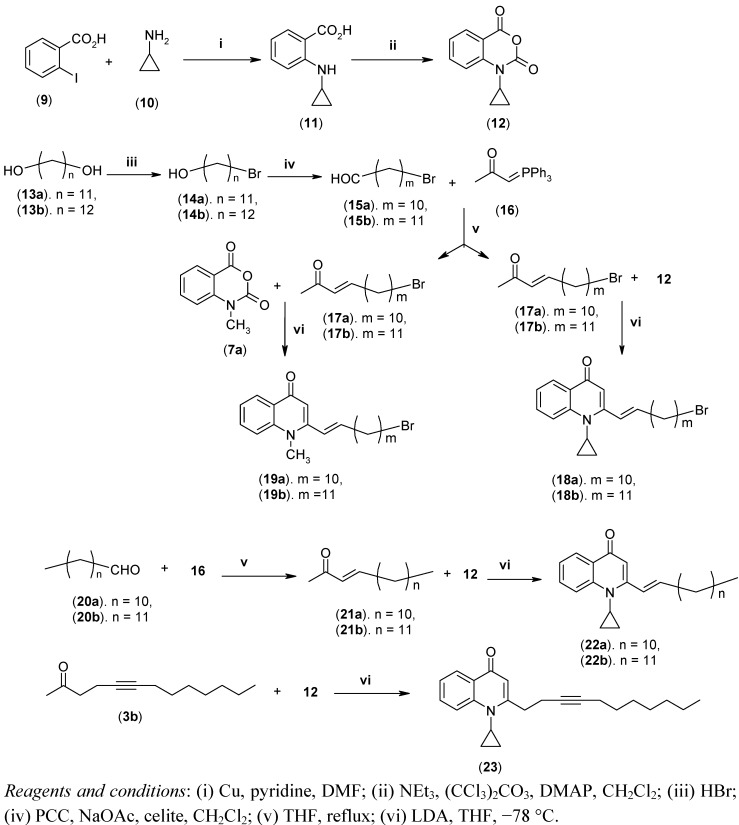
Synthesis of *N*-cyclopropyl-2-(*E*)-alkenyl-4(1*H*)-quinolones and *N*-cyclo- propyl-2-alkynyl-4(1*H*)-quinolones.

In order to study the biological effect of the cyclopropyl moiety which is the constituent of many therapeutically important fluoroquinolones, we explored the possibility of introducing a cyclopropyl group at *N*-1. *N*-Cyclopropyl isatoic acid anhydride (**12**) was synthesized from 2-iodobenzoic acid (**9**) as shown in Scheme 2. Copper (0) mediated substitution of the iodine of **9** with cyclopropyl amine in DMF gave 2-(cyclopropylamino)benzoic acid (**11**). Compound **11** was then treated with NEt_3_ and bis-(trichloromethyl) carbonate in the presence of catalytic *N,N*-dimethylaminopyridine in CH_2_Cl_2_ to afford **12**.

The intermediate, ω-bromo-α,β-unsaturated methyl alkenyl ketones **17a–b**, required to incorporate a terminal bromine atom in the aliphatic side chain, were prepared from commercially available α,ω-diols **13a–b**. Bromination of the diols with HBr and subsequent oxidation with pyridinium chlorochromate (PCC) gave ω-bromoaldehydes **15a–b**. Compounds **17a–b** were prepared as previously reported [11] by refluxing **15a–b** with the ylide methylcarbonylmethylenephosphorane (**16**) obtained from chloroacetone and triphenylphosphine.

To further assess the biological role of the cyclopropyl moiety at *N*-1 and compare their antimycobacterial properties with our previously reported *N*-methyl-2-(*E*)-alkenyl-4(1*H*)-quinolones, compounds **22a–b** were prepared from the commercially available aldehydes **20a–b** following the same methods described before [10]. Similarly, analogue **23** containing cyclopropyl and undecynyl groups at positions 1 and 2, respectively (Scheme 2), was prepared from compounds **3b** and **12** in order to examine their impact on biological potency. The identity of the synthetic intermediates and the 4(1*H*)-quinolone derivatives was confirmed by analysis of 1D and 2D-NMR spectroscopic and LC-ESI-MS data. The compounds **8a–8t**, **18a–b**, **19a–b**, **22a–b** and **23** are reported here for the first time.

### 2.2. Biological Evaluation

All of the novel synthetic compounds were screened for their *in vitro* antimycobacterial activity against *M. smegmatis* using a microbroth dilution method [10] and the most active compounds were further tested against *M. fortuitum*, *M. phlei*, *M. smegmatis* mc^2^155 and *M. bovis* BCG. Enzymatic inhibition of the MurE ligase of *M. tuberculosis* was also determined.

In our microtiter broth dilution assay, the quinolone derivatives displayed significant inhibitory effect against *M. smegmatis*, with MIC values ranging from 0.5 to 128 mg/L (1.2–350.7 µM). The most active compounds were **8n**, **8r**, **19a** and **19b**, with an MIC value of 0.5 mg/L (1.2–1.5 µM). 

As shown in Table 1, compound **8a** with the shortest aliphatic chain (3-decynyl and methyl groups at position 2 and 1, respectively), displayed the lowest growth inhibition (MIC value of 32 mg/L). As the aliphatic chain length increased, an increase in potency was observed with maximum activity achieved for 1-tetradecynyl (compound **8r**) and 1-dodecynyl (compound **8n**) groups. Further elongation of the aliphatic chain resulted in a gradual loss of activity. The antimycobacterial data in Table 1 also indicated that the potency of the quinolone derivatives was related to chain length of the aliphatic group at position 1 and 2. As shown in Table 2, among the rapidly-growing strains of mycobacteria, *M. smegmatis* mc^2^155 was found to be the most susceptible to the test compounds. In the spot culture growth inhibition assay (SPOTi), the tested compounds were found to be moderate growth inhibitors of *M. bovis* BCG (MIC value of 25 mg/L). Against the epidemic strains of MRSA (EMRSA-15 and -16) the most active quinolone derivatives, **8l**, **8m** and **8n** displayed pronounced growth inhibition with MIC values ranging from 2 to 8 mg/L (5.5–22.8 µM). 

**Table 1:** MIC values against *M. smegmatis* and cytotoxicity against MRC-5 cells of the *N*-alkyl-2-alkynyl/(*E*)-alkenyl-4(1*H*)-quinolone derivatives. 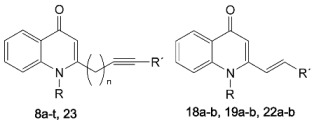

Comp.	Substituent			MIC against *M. smegmatis* (ATCC 14468)		Metabolic active MRC-5 cells (%)			
	n	R	R'	mg/L	µM	100 µM	60 µM	30 µM	10 µM
**8a**	2	–CH_3_	hexyl	32	108.5	89.3 ± 2.94	97.5 ± 0.74	98.4 ± 1.07	100 ± 1.05
**8b**	2	–C_2_H_5_	hexyl	16	51.8	85.7 ± 1.96	98.4 ± 0.77	100 ± 0.73	102 ± 0.85
**8c**	2	–C_3_H_7_	hexyl	8	24.8	100 ± 1.45	100 ± 0.55	102 ± 0.42	103 ± 1.22
**8d**	2	–CH_3_	heptyl	8	25.9	97.3 ± 0.66	98.7 ± 0.8	99.8 ± 0.52	101 ± 1.08
**8e**	2	–C_2_H_5_	heptyl	8	24.8	100 ± 1.05	100 ± 0.56	101 ± 1.42	102 ± 1.05
**8f**	2	–C_3_H_7_	heptyl	4	11.9	70.9 ± 8.50	91.7 ± 2.77	101 ± 1.28	100 ± 1.05
**8g**	0	–C_2_H_5_	octyl	2	6.5	12.1 ± 4.79	56.5 ± 2.03	98.7 ± 2.97	101 ± 0.97
**8h**	0	–C_3_H_7_	octyl	4	12.4	14.1 ± 7.83	39.5 ± 3.59	101 ± 2.47	103 ± 1.38
**8i**	0	C_4_H_9_	octyl	4	11.9	28.7 ± 8.95	67.2 ± 9.90	101 ± 0.81	103 ± 3.13
**8j**	2	–CH_3_	octyl	4	12.4	95.84 ± 0.96	95.1 ± 0.81	96.7 ± 1.49	97.6 ± 1.03
**8k**	2	–C_2_H_5_	octyl	4	11.9	74.8 ± 1.99	94.7 ± 0.92	95.1 ± 1.04	97.6 ± 1.69
**8l**	2	–C_3_H_7_	octyl	1	2.8	9.0 ± 6.85	69.1 ± 8.04	99.1 ± 2.32	100 ± 0.31
**8m**	2	–C_4_H_9_	octyl	2	5.5	0.4 ± 0.02	67.5 ± 3.19	89.7 ± 2.46	94.9 ± 3.18
**8n**	0	–CH_3_	decyl	0.5	1.5	100 ± 0.82	100 ± 0.81	100 ± 0.72	101 ± 0.85
**8o**	2	–CH_3_	decyl	2	5.7	90.7 ± 3.48	95.8 ± 2.54	97.9 ± 0.88	100 ± 0.73
**8p**	2	–C_2_H_5_	decyl	1	2.7	8.75 ± 9.35	93.3 ± 1.13	95.4 ± 0.76	97.3 ± 1.37
**8q**	2	–C_3_H_7_	decyl	1	2.6	0.2 ± 0.09	19.8 ± 9.85	67.9 ± 2.65	92.7 ± 4.62
**8r**	0	–CH_3_	dodecyl	0.5	1.5	95.7 ± 1.14	97.1 ± 1.69	98.1 ± 1.65	99.7 ± 2.03
**8s**	2	–CH_3_	dodecyl	128	337.7	41.5 ± 9.49	58.9 ± 3.65	92.2 ± 0.70	93.9 ± 0.61
**8t**	0	–CH_3_	tridecyl	128	350.7	93.8 ± 0.96	98.8 ± 1.28	100 ± 1.38	100 ± 1.01
**18a**	-	Cp	bromodecyl	8	18.6	ND	ND	ND	ND
**18b**	-	Cp	bromoundecyl	8	18.1	ND	ND	ND	ND
**19a**	-	–CH_3_	bromodecyl	0.5	1.2	ND	ND	ND	ND
**19b**	-	–CH_3_	bromoundecyl	0.5	1.2	ND	ND	ND	ND
**22a**	-	Cp	undecyl	128	350.7	ND	ND	ND	ND
**22b**	-	Cp	dodecyl	128	337.8	ND	ND	ND	ND
**23**	2	Cp	hexyl	8	24.3	ND	ND	ND	ND
**Isoniazid**	-	-	-	4	29.2	ND	ND	ND	ND
**Ethambutol**	-	-	-	2	9.8	ND	ND	ND	ND
**Vinblastin** *						51.5 ± 2.36			

ND: not determined; * 0.12 µM.

**Table 2:** *In vitro* biological activity of selected *N*-alkyl-4(1*H*)-quinolone derivatives and positive controls.

Compound	MIC mg/L(µM)						IC_50_ (µM)
	*M. smegmatis* mc^2^155	*M. fortuitum*	*M. phlei*	*M. bovis* BCG	*S. aureus* EMRSA-15	*S. aureus* EMRSA-16	*Mtb* MurE
**8g**	4 (12.9)	8 (25.9)	4 (12.9)	ND	ND	ND	ND
**8l**	2 (5.7)	2 (5.7)	4 (11.4)	25 (71.2)	2 (5.7)	8 (22.8)	263
**8m**	2 (5.5)	2 (5.5)	4 (10.9)	25 (68.5)	2 (5.5)	4 (10.9)	235
**8n**	1 (3.1)	0.5 (1.5)	1 (3.1)	25 (77.4)	2 (6.2)	4 (12.4)	251
**8o**	1 (2.8)	2 (5.7)	2 (5.7)	25 (71.2)	8 (22.8)	8 (22.8)	774
**8p**	1 (2.7)	4 (10.9)	2 (5.5)	ND	ND	ND	ND
**8q**	1 (2.6)	2 (5.3)	2 (5.3)	25 (66.0)	2 (5.3)	128 (337.7)	200
**8r**	1 (2.8)	0.5 (1.5)	2 (5.7)	25 (71.2)	16 (45.6)	128 (364.7)	631
**19a**	1 (2.5)	1 (2.5)	1 (2.5)	ND	ND	ND	ND
**19b**	1 (2.4)	1 (2.4)	1 (2.4)	ND	ND	ND	ND
**Isoniazid**	4 (29.2)	2 (14.6)	8 (58.3)	0.1 (0.7)	ND	ND	ND
**Ethambutol**	4 (19.6)	8 (39.2)	4 (19.6)	ND	ND	ND	ND
**Norfloxacin**	ND	ND	ND	ND	0.5 (1.6)	128 (400.9)	ND
**Tetracycline**	ND	ND	ND	ND	0.25 (0.6)	0.25 (0.6)	ND
**1-Methyl-2-[(5*Z*)-tetradecenyl]-4(1*H*)-quinolone**	ND	ND	ND	ND	ND	ND	36 [7]

ND: Not determined.

When assayed against *M. tuberculosis* ATP-dependent MurE using a colorimetric method the tested compounds displayed moderate to low activity, with IC_50_ values ranging from 200 µM to 774 µM. Our biological results further strengthen our previous finding that optimal activity is achieved when the 4(1*H*)-quinolones possess an aliphatic chain at C-2 which is 12–14 carbons in length. When the length of the aliphatic side chain at position 2 of the quinolone nucleus was further increased, for instance compounds **8s** and **8t**, a marked reduction of the antimycobacterial activity was noted. A significant decrease in antimycobacterial potency was also encountered upon introduction of a cyclopropyl group at N-1. However, it should be pointed out that compounds **8c** and **23**, both having a dec-3-ynyl group at position 2 of the quinolone nucleus and *n*-propyl and cyclopropyl groups at N-1, respectively, were found to be equipotent against *M. smegmatis*. The most active compounds **8n** and **8r** are of particular interest because they are devoid of toxicity to the human diploid embryonic lung cell line (MRC-5) up to a concentration of 100 µM.

The antimycobacterial activity of compounds having the same substituent at N-1 and an alkynyl group with the same chain length at position 2, but differing on the position of the triple bond, such as **8b** and **8g** (*N*-Et and decynyl), **8c** and **8h** (*N*-propyl and decynyl), **8j** and **8n** (*N*-Me and dodecynyl) and **8o** and **8r** (*N*-Me and tetradecynyl), was compared in order to assess the effect of the position of the triple bond on the activity. It was realized that compounds **8g**, **8h**, **8n** and **8r** exhibited two to four fold greater potencies than **8b**, **8c**, **8j** and **8o**. In general, a higher antimycobacterial activity was observed for compounds having the triple bond in position α,β to the quinolone nucleus compared to compounds having the triple bond in a position distant from the quinolone nucleus. However, this tendency was inverted for *N*-propyl or *N*-cyclopropyl substitution, where compounds having the triple bond distant from the quinolone **8l** and **8m** were slightly more active than compounds having the triple bond in position α,β, such as **8h** and **8i**. This SAR suggests that antimycobacterial potency depends on different structural parameters in a non-linear way and a relation valid for a set of compounds might not be valid for a different set, and thus point out on a complementary approach for obtaining meaningful SAR.

Compounds **18a–b** and **19a–b** were prepared in order to explore the effects of a terminal bromine atom on the antimycobacterial activity. It is worth mentioning that compound **19b** showed a two-fold increase in antimycobacterial potency against *M. smegmatis* compared to its analogue lacking a bromine atom reported in our previous work [10,11]. This result is quite interesting and we are aiming to explore the possibility of the same effect for other active anti-TB scaffolds having long aliphatic chains. The presence of a bromine at the terminal position may increase the probability of covalent bonding to a particular protein by nucleophilic attack, an hypothesis which deserves further attention. 

On the other hand, compounds **18a–b** and **22a–b**, bearing a cyclopropyl moiety at *N*-1 appeared to be less potent than their analogues **19a–b** having a methyl group at *N*-1, again suggesting that a cyclopropyl group is detrimental to the activity.

In order to assess the cytotoxicity of these novel quinolone derivatives, the effect on the viability of a human lung fibroblast cell line MRC-5, was measured using XTT proliferation assay. The compounds displayed little or no toxicity at 100 µM test concentration. By comparing the cytotoxicity results of this study with that of our previous reports [10,11], one can notice a remarkable reduction in cytotoxicity to the MRC-5 cell line, indicating that the triple bond was important in reducing the toxicity of the quinolones. This finding finds support in other studies which revealed that 5-decynyl, 5-dodecynyl and 5-tetradecynyl pyrimidine nucleoside derivatives have enhanced antimycobacterial activity while being devoid of cytotoxic activity up to a high concentration [15]. In addition, the position of the triple bond in the side chain seems to have a significant influence on cytotoxcity which is increased in compounds with a triple bond more distant to the quinolone nucleus. This can be concluded by comparing results of **8l**, **8n**, **8p**, and **8r** having similar antimycobacterial activity on *M. smegmatis* but different cytotoxcity depending on triple bond position.

## 3. Experimental

### 3.1. General

The strains *M. smegmatis* (ATCC 14468), *M. smegmatis* mc^2^155, *M. fortuitum* (ATCC 6841), *M. phlei* (ATCC 11758) and *M. bovis* BCG (ATCC 35734) were obtained from American Type Culture Collection or German Collection of Microorganisms and Cell Culture (DSMZ). EMRSA-15 and 16 were gifts from Dr Paul Stapleton (UCL School of Pharmacy, UK). *M. tuberculosis* MurE ligase was over-expressed in *E. coli* BL21(DE3)pLysS cells (New England Biolabs, Hitchin, UK) and purified according to our previous report [7].

All chemicals were purchased from Sigma-Aldrich (Munich, Germany). Reactions were carried out using oven-dried glassware under an atmosphere of argon. THF was distilled from sodium and stored in molecular sieve (4 Å). DMF was distilled from CaH_2_. Melting points were recorded using a KOFLER hot plate microscope and are uncorrected. IR spectra obtained on a Perkin-Elmer 281 B spectrometer, were recorded in KBr unless otherwise noted. ^1^H and ^13^C-NMR spectra were recorded on a Varian 400 MHz spectrometer (at 400 and 100 MHz, respectively) using deuterated chloroform as solvent with TMS as internal standard. Mass spectra were obtained by LC-ESI-MS analysis in positive mode on a Thermo Finnigan LCQ Deca XP Plus mass spectrometer connected to a Surveyor LC-system (Thermo-Finnigan, West Palm Beach, FL, USA). Precoated Si gel 60 F_254_ plates (Merck, Darmstadt, Germany) were used to monitor the progress of the reactions and column fractions. Spots were detected by UV/254 nm and spraying with molybdato-phosphoric acid and subsequent heating. Compounds were purified by column chromatography on silica gel 60 (0.063–0.200 mm) using cyclohexane/ethyl acetate mixtures as eluent.

### 3.2. Synthesis

#### 3.2.1. General Procedure for Synthesis of Alkynones **3a–e**

Compounds **3a–e** were obtained according to the procedure described previously [13] with some modifications. To a stirred solution of Pd(OAc)_2_ (0.05 equiv.) in P(CH_3_)_3_ (1.0 M in toluene, 0.2 equiv.) at 60 °C, acetone (~2 mL) and a mixture of 1-alkyne **1** (1 equiv.) and methyl vinyl ketone (**2**, 3.0 equiv.) were added. The reaction mixture was stirred for 24 h at 60 °C. Then the catalyst was filtered off on celite, extracted with ether, dried over Na_2_SO_4_ and concentrated. The crude product was purified by silica gel CC eluting with cyclohexane/ethyl acetate = 95:5 to afford **3a–e**.

*5-Dodecyn-2-one* (**3a**) was prepared from 1-octyne (5.0 g, 45.5 mmol), **2** (9.6 g, 136.4 mmol), Pd(OAc)_2_ (0.51 g, 2.3 mmol) and P(CH_3_)_3_ (9.1 mL, 9.1 mmol) as a colourless oil (75%) [16].

*5-Tridecyn-2-one* (**3b**) was prepared from 1-nonyne (5.0 g, 40.3 mmol), **2** (8.5 g, 121 mmol), Pd(OAc)_2_ (0.45 g, 2.0 mmol) and P(CH_3_)_3_ (8.1 mL, 8.1 mmol) as a colourless oil (82%). ^1^H-NMR *δ*: 2.60 (t, *J* = 7.6 Hz, 2H, H-3), 2.39 (t, *J* = 7.6 Hz, 2H, H-4), 2.16 (s, 3H, H-1), 2.10 (t, *J* = 7.6 Hz, 2H, H-7), 1.43 (quint, *J* = 6.8 Hz, 2H, H-8), 1.30–1.25 (m, 8H, H-9-12), 0.86 (t, *J* = 6.8 Hz, 3H, H-13). ^13^C-NMR *δ*: 207.2 (C-2), 80.7 (C-6), 78.4 (C-5), 43.0 (C-3), 31.8, 29.4, 29.3, 29.1, 28.9, 22.6, 18.6, 14.0, 13.5.

*5-Tetradecyn-2-one* (**3c**) was prepared from 1-decyne (5.0 g, 36.2 mmol), **2** (7.6 g, 108.7 mmol), Pd(OAc)_2_ (0.41 g, 1.8 mmol) and P(CH_3_)_3_ (7.2 mL, 7.2 mmol) as a colourless oil (79%) [13].

*5-Hexadecyn-2-one* (**3d**) was prepared from 1-dodecyne (5.0 g, 30.1 mmol), **2** (6.3 g, 90.4 mmol), Pd(OAc)_2_ (0.34 g, 1.5 mmol) and P(CH_3_)_3_ (6.0 mL, 6.0 mmol) as a colourless oil (73%). ^1^H-NMR *δ*: 2.61 (t, *J* = 7.2 Hz, 2H, H-3), 2.40 (t, *J* = 7.2 Hz, 2H, H-4), 2.15 (s, 3H, H-1), 2.09 (t, *J* = 7.2 Hz, 2H, H-7), 1.43 (quint, *J* = 6.8 Hz, 2H, H-8), 1.30–1.23 (m, 14H, H-9-15), 0.87 (t, *J* = 6.8 Hz, 3H, H-16). ^13^C-NMR *δ*: 207.1 (C-2), 80.9 (C-6), 78.3 (C-5), 42.9 (C-3), 31.9, 29.8, 29.5, 29.5, 29.3, 29.1, 29.0, 28.8, 22.6, 18.6, 14.0, 13.5.

*5-Octadecyn-2-one* (**3e**) was prepared from 1-tetradecyne (4.0 g, 20.6 mmol), **2** (4.3 g, 61.9 mmol), Pd(OAc)_2_ (0.23 g, 1.0 mmol) and P(CH_3_)_3_ (4.1 mL, 4.1 mmol) as a colourless oil (73%). ^1^H-NMR *δ*: 2.59 (t, *J* = 7.6 Hz, 2H, H-3), 2.41 (t, *J* = 7.6 Hz, 2H, H-4), 2.14 (s, 3H, H-1), 2.08 (t, *J* = 7.6 Hz, 2H, H-7), 1.44 (quint, *J* = 6.8 Hz, 2H, H-8), 1.33–1.21 (m, 18H, H-9-17), 0.85 (t, *J* = 6.8 Hz, 3H, H-18). ^13^C-NMR *δ*: 207.3 (C-2), 81.0 (C-6), 78.5 (C-5), 42.8 (C-3), 31.8, 29.8, 29.8, 29.5, 29.5, 29.4, 29.3, 29.1, 29.0, 28.8, 22.6, 18.6, 14.0, 13.5.

#### 3.2.2. General Procedure for Synthesis of Alkynols **5a–d**

To a stirred solution of 1-alkyne (1 equiv.) in THF at −78 °C, *n*-BuLi (1.6 M in hexane) (1.1 equiv.) was added dropwise and stirring was continued for 2 h. To this solution acetaldehyde (2.0 equiv.) was added and further stirred for about 1 h until the temperature dropped to room temperature. The reaction was quenched by the addition of NH_4_Cl solution and extracted with CH_2_Cl_2_ (3×), washed with brine, dried over Na_2_SO_4_ and concentrated. The crude product was purified by silica gel CC eluting with cyclohexane/ethyl acetate = 9:1.

*3-Dodecyn-2-ol* (**5a**) was prepared from 1-decyne (10.0 g, 72.5 mmol) in THF (150 mL), *n*-BuLi (49.8 mL, 79.7 mmol), acetaldehyde (6.4 g, 145.1 mmol) as a yellow oil (77%). ^1^H-NMR *δ*: 4.46 (qd, *J* = 6.4, 1.6 Hz, 1H, H-2), 2.14 (td, *J* = 6.8, 1.6 Hz, 2H, H-5), 1.45 (quint, *J* = 7.6 Hz, 2H, H-6), 1.37 (d, *J* = 6.4 Hz, 3H, H-1), 1.30–1.21 (m, 10H, H-7-11), 0.85 (t, *J* = 6.8 Hz, 3H, H-12). ^13^C-NMR *δ*: 84.4 (C-4), 82.2 (C-3), 58.4 (C-2), 31.2, 29.1, 29.0, 28.5, 28.4, 24.6, 22.4, 18.5, 13.9.

*3-Tetradecyn-2-ol* (**5b**) was prepared from 1-dodecyne (4.0 g, 24.1 mmol) in THF (75 mL), *n*-BuLi (16.6 mL, 26.5 mmol), acetaldehyde (2.1 g, 48.2 mmol) as a yellow oil (69%) [17].

*3-Hexadecyn-2-ol* (**5c**) was prepared from 1-tetradecyne (5.0 g, 25.8 mmol) in THF (80 mL), *n*-BuLi (17.7 mL, 28.4 mmol), acetaldehyde (2.27 g, 51.6 mmol) as a yellow oil (81%) [18]. 

*3-Heptadecyn-2-ol* (**5d**) was prepared from 1-pentadecyne (4.0 g, 19.2 mmol) in THF (75 mL), *n*-BuLi (13.2 mL, 21.2 mmol), acetaldehyde (1.69 g, 38.5 mmol) as a yellow oil (65%). ^1^H-NMR *δ*: 4.49 (qd, *J* = 6.8, 1.6 Hz, 1H, H-2), 2.17 (td, *J* = 6.8, 1.6 Hz, 2H, H-5), 1.48 (quint, *J* = 7.2 Hz, 2H, H-6), 1.40 (d, *J* = 6.4 Hz, 3H, H-1), 1.33–1.21 (m, 20H, H-7-16), 0.87 (t, *J* = 6.8 Hz, 3H, H-17). ^13^C-NMR *δ*: 84.7 (C-4), 82.2 (C-3), 58.5 (C-2), 31.9, 29.7, 29.6, 29.6, 29.6, 29.5, 29.3, 29.1, 28.8, 28.6, 24.7, 22.6, 18.6, 14.1.

#### 3.2.3. General Procedure for the Synthesis of Alkynones **6a–d**

A mixture of **5** (1.0 equiv.) in THF and MnO_2_ (0.67 equiv.) was stirred at room temperature for 24 h. The reaction mixture was filtered through celite to give **6**.

*3-Dodecyn-2-one* (**6a**) was prepared from **5a** (10.0 g, 54.9 mmol), in THF (50 mL) and MnO_2_ (3.2 g, 36.8 mmol) as a colorless oil (79%). ^1^H-NMR *δ*: 2.32 (t, *J* = 7.2 Hz, 2H, H-5), 2.29 (s, 3H, H-1), 1.55 (quint, *J* = 7.6 Hz, 2H, H-6), 1.30–1.23 (m, 10H, H-7-11), 0.87 (t, *J* = 6.8 Hz, 3H, H-12). ^13^C-NMR *δ*: 184.8 (C-2), 94.1 (C-4), 81.3 (C-3), 32.7, 31.1, 29.3, 29.0, 28.4, 27.6, 22.5, 18.9, 14.0.

*3-Tetradecyn-2-one* (**6b**) was prepared from **5b** (3.0 g, 14.3 mmol), in THF (15 mL) and MnO_2_ (0.83 g, 9.6 mmol) as a colourless oil (87%). ^1^H-NMR *δ*: 2.33 (t, *J* = 6.8 Hz, 2H, H-5), 2.30 (s, 3H, H-1), 1.56 (quint, *J* = 7.2 Hz, 2H, H-6), 1.31–1.23 (m, 14H, H-7-13), 0.87 (t, *J* = 6.8 Hz, 3H, H-14). ^13^C-NMR *δ*: 184.8 (C-2), 94.1 (C-4), 81.4 (C-3), 32.7, 31.8, 29.5, 29.4, 29.2, 29.0, 28.8, 27.6, 22.6, 18.9, 14.0.

*3-Hexadecyn-2-one* (**6c**) was prepared from **5c** (4.5 g, 18.9 mmol), in THF (20 mL) and MnO_2_ (1.1 g, 12.7 mmol) as a colourless oil (82%). ^1^H-NMR *δ*: 2.32 (t, *J* = 6.8 Hz, 2H, H-5), 2.29 (s, 3H, H-1), 1.55 (quint, *J* = 7.2 Hz, 2H, H-6), 1.33–1.19 (m, 18H, H-7-15), 0.86 (t, *J* = 6.8 Hz, 3H, H-16). ^13^C-NMR *δ*: 184.7 (C-2), 94.0 (C-4), 81.3 (C-3), 32.6, 31.8, 29.6, 29.6, 29.5, 29.4, 29.2, 29.0, 28.8, 27.6, 22.6, 18.9, 14.0.

*3-Heptadecyn-2-one* (**6d**) was prepared from **5d** (3.0 g, 11.9 mmol), in THF (15 mL) and MnO_2_ (0.69 g, 7.9 mmol) as a colourless oil (83%). ^1^H-NMR *δ*: 2.34 (t, *J* = 6.8 Hz, 2H, H-5), 2.31 (s, 3H, H-1), 1.57 (quint, *J* = 7.6 Hz, 2H, H-6), 1.33–1.21 (m, 20H, H-7-16), 0.87 (t, *J* = 6.8 Hz, 3H, H-17). ^13^C-NMR *δ*: 184.9 (C-2), 94.1 (C-4), 81.4 (C-3), 32.7, 31.9, 29.6, 29.6, 29.6, 29.6, 29.4, 29.3, 29.0, 28.8, 27.7, 22.7, 18.9, 14.1.

#### 3.2.4. Preparation of 2-(Cyclopropylamino)benzoic acid (**11**)

2-Iodobenzoic acid (10.0 g, 40.3 mmol, 1.0 equiv.) dissolved in DMF (50 mL) was poured into an argon purged two mouth flask containing freshly prepared Cu (512 mg, 8.1 mmol, 0.2 equiv.), pyridine (4.8 g, 60.5 mmol, 1.5 equiv.) and cyclopropylamine (5.1 g, 88.7 mmol, 2.2 equiv.). The mixture was stirred for 24 h at room temperature and poured into 500 mL of acidified water (pH 4.5). Filtration and drying of the slurry gave a white precipitate of **11** (83% yield). ^1^H-NMR *δ*:7.98 (d, *J* = 8.0 Hz, 1H, H-6), 7.72 (bs, 1H, –NH–), 7.44 (t, *J* = 7.6 Hz, 1H, H-4), 7.17 (d, *J* = 8.0 Hz, 1H, H-3), 6.68 (t; *J* = 7.6 Hz, 1H, H-5), 2.49 (sept, *J* = 3.2 Hz, 1H, N–CH–), 0.83 (m, 2H, N–CH–CH_2_–), 0.60 (m, 2H, N–CH–CH_2_–). ^13^C-NMR *δ*: 174.0 (–COOH), 152.4 (C-2), 135.4 (C-4), 132.3 (C-6), 115.3 (C-5), 112.9 (C-3), 108.8 (C-1), 24.2 (N–CH–), 7.6 (2 × N–CH–CH_2_–).

#### 3.2.5. Preparation of N-Cyclopropylisatoic Anhydride (**12**)

2-(Cyclopropylamine)benzoic acid (4.0 g, 22.6 mmol, 1 equiv.) and N(Et)_3_ (2.2 g, 21.5 mmol, 0.95 equiv.) were dissolved in CH_2_Cl_2_ (50 mL) and cooled to 0 °C with ice. Bis(trichloromethyl) carbonate (2.0 g, 6.8 mmol, 0.3 equiv.) dissolved in CH_2_Cl_2_ (25 mL) was added to the mixture using a syringe followed by *N,N*-dimethylaminopyridine (0.4 g, 3.3 mmol, 0.15 equiv.) in CH_2_Cl_2_ (15 mL). After 2 h of stirring at the same temperature the reaction was quenched by adding 25 mL of HCl (1.0 M), extracted with CH_2_Cl_2_, dried over Na_2_SO_4_ and concentrated to give a light yellow powder of **12** (94%). ^1^H-NMR *δ*: 8.11 (d, *J* = 8.0 Hz, 1H, H-5), 7.79 (t, *J* = 7.6 Hz, 1H, H-7), 7.65 (d, *J* = 8.0 Hz, 1H, H-8), 7.31 (t, *J* = 7.6 Hz, 1H, H-6), 2.93 (quint, *J* = 3.2 Hz, 1H, N–CH–), 1.33 (m, 2H, N–CH–CH_2_–), 0.99 (m, 2H, N–CH–CH_2_–). ^13^C-NMR *δ*: 158.7 (C-4), 147.7 (C-2), 142.6 (C-8a), 136.8 (C-7), 130.3 (C-5), 124.1 (C-6), 115.4 (C-8), 111.8 (4a), 27.1 (N–CH–), 9.9 (2 × N–CH–CH_2_–).

#### 3.2.6. Synthesis of ω-Bromoalcohols **14a–b**

Treatment of the diols **13a–b** with 48% of HBr according to a previously described method [10] provided 11-bromoundecan-1-ol (**14a**) [19] and 12-bromododecan-1-ol (**14b**) [20].

#### 3.2.7. Synthesis of ω-Bromoaldehydes **15a–b**

Both 11-bromoundecanal (**15a**) and 12-bromodecanal were obtained from **14a** and **14b**, respectively, by the action of PCC in the presence of NaOAc in CH_2_Cl_2_ according to Houghton *et al.* [21], spectral data are in accordance with [22].

#### 3.2.8. Synthesis of ω-Bromo-(3*E*)-ketones **17a–b**

*14-Bromo-(3E)-tetradecen-2-one* (**17a**) was prepared from **15a** and ylide **16** according to Wube *et al.* [10] as a colorless oil (78% yield). ^1^H-NMR *δ*: 6.81 (dt, *J* = 16.0, 6.8 Hz, 1H, H-4), 6.06 (d, *J* = 16.0 Hz, 1H, H-3), 3.41 (t, *J* = 6.8 Hz, 2H, H-14), 2.25 (s, 3H, H-1), 2.21 (q, *J* = 7.2 Hz, 2H, H-5), 1.84 (quint, *J* = 6.8 Hz, 2H, H-13), 1.44 (m, 4H, H-12, 6), 1.32–1.23 (m, 10H, H-7-11). ^13^C-NMR *δ*: 198.6 (C-2), 148.5 (C-4), 131.1 (C-3), 33.9 (C-14), 32.6 (C-13), 32.3 (C-5), 29.2 (C-10), 29.2 (C-9), 29.0 (C-8), 28.6 (C-6), 28.0 (C-7), 27.9 (C-11), 26.7 (C-12), 26.7 (C-1).

*15-Bromo-(3E)-pentadecen-2-one* (**17b**) was prepared from **15b** and ylide **16** as a colourless oil (84% yield). ^1^H-NMR *δ*: 6.81 (dt, *J* = 16.0, 6.4 Hz, 1H, H-4), 6.06 (d, *J* = 16.0 Hz, 1H, H-3), 3.41 (t, *J* = 6.8 Hz, 2H, H-15), 2.25 (s, 3H, H-1), 2.22 (q, *J* = 6.8 Hz, 2H, H-5), 1.85 (quint, *J* = 6.8 Hz, 2H, H-14), 1.43 (m, 4H, H-13, 6), 1.32–1.23 (m, 12H, H-7-12). ^13^C-NMR *δ*: 198.9 (C-2), 148.7 (C-4), 131.2 (C-3), 33.9 (C-15), 32.4 (C-14), 32.4 (C-5), 29.4 (C-10), 29.4 (C-9), 29.3 (C-11), 29.3 (C-8), 29.1 (C-7), 28.6 (C-6), 28.0 (C-12), 26.8 (C-13), 26.7 (C-1).

#### 3.2.9. General Procedure for the Synthesis of **8a–t**, **18a–b**, **19a–b**, **22a–b** and **23**

Compounds **8a–t**, **18a–b**, **19a–b**, **22a–b** and **23** were prepared according to procedure described previously [10] from methyl alkynyl ketones (1.0 equiv.) in THF, LDA (1.8 M in THF/heptane/ethylbenzene) (1.0 equiv.) and *N*-alkyl isatoic anhydride (0.75 equiv.) in THF at −78 °C. The purity of the quinolones was determined by LC-MS (88–95%).

*1-Methyl-2-(3′-decynyl)-4(1H)-quinolone* (**8a**) was prepared from **3a** (1.0 g, 5.6 mmol) in THF (15 mL), LDA (3.1 mL, 5.6 mmol) and *N*-methylisatoic anhydride (**7a**) (0.74 g, 4.2 mmol) in THF (10 mL) as a yellow oil (63%). IR (KBr, cm^−1^): 3406, 2929, 2857, 1627, 1600, 1500, 1469, 1177, 759. ^1^H-NMR *δ*: 8.44 (d, *J* = 8.0 Hz, 1H, H-5), 7.68 (t, *J* = 7.6 Hz, 1H, H-7), 7.52 (d, *J* = 8.0 Hz, 1H, H-8), 7.39 (t, *J* = 7.2 Hz, 1H, H-6), 6.32 (s, 1H, H-3), 3.78 (s, 3H, N–CH_3_), 2.95 (t, *J* = 7.2 Hz, 2H, H-1'), 2.57 (t, *J* = 6.8 Hz, 2H, H-2'), 2.12 (t, *J* = 6.8 Hz, 2H, H-5'), 1.44 (quint, *J* = 7.2 Hz, 2H, H-6'), 1.32–1.20 (m, 6H, H-7'-9'), 0.86 (t, *J* = 6.8 Hz, 3H, H-10'). ^13^C-NMR *δ*: 177.5 (C-4), 152.6 (C-2), 140.6 (C-8a), 132.2 (C-7), 126.8 (C-5), 126.6 (C-4a), 123.5 (C-6), 115.5 (C-8), 110.7 (C-3), 82.8 (C-4'), 77.2 (C-3'), 35.3 (N–CH_3_), 32.9 (C-1'), 31.6 (C-8'), 28.6 (C-7'), 28.4 (C-6'), 22.6 (C-9'), 18.7 (C-2'), 18.5 (C-5'), 14.0 (C-10'). ESI-MS *m/z* (rel. int.): [M+H]^+^ 296 (100).

*1-Ethyl-2-(3'-decynyl)-4(1H)-quinolone* (**8b**) was prepared from **3a** (1.0 g, 5.6 mmol) in THF (15 mL), LDA (3.1 mL, 5.6 mmol) and *N*-ethylisatoic anhydride (**7b**) (0.8 g, 4.2 mmol) in THF (10 mL) as a yellow oil (55%). IR (KBr, cm^−1^): 3428, 2929, 2854, 1624, 1600, 1490, 1468, 1430, 1308, 759. ^1^H-NMR *δ*: 8.46 (d, *J* = 8.0 Hz, 1H, H-5), 7.63 (t, *J* = 7.6 Hz, 1H, H-7), 7.55 (d, *J* = 8.0 Hz, 1H, H-8), 7.37 (t, *J* = 7.6 Hz, 1H, H-6), 6.34 (s, 1H, H-3), 4.33 (q, *J* = 7.2 Hz, 2H, N–CH_2_–CH_3_), 2.96 (t, *J* = 7.2 Hz, 2H, H-1'), 2.61 (t, *J* = 6.8 Hz, 2H, H-2'), 2.13 (t, *J* = 6.8 Hz, 2H, H-5'), 1.45 (t, *J* = 7.2 Hz, 3H, N–CH_2_–CH_3_), 1.42 (quint, *J* = 6.8 Hz, 2H, H-6'), 1.30–1.21 (m, 6H, H-7'-9'), 0.86 (t, *J* = 6.8 Hz, 3H, H-10'). ^13^C-NMR *δ*: 177.4 (C-4), 152.6 (C-2), 140.5 (C-8a), 132.2 (C-7), 126.9 (C-5), 126.7 (C-4a), 123.4 (C-6), 115.4 (C-8), 110.9 (C-3), 82.7 (C-4'), 77.1 (C-3'), 41.2 (N–CH_2_–CH_3_), 33.0 (C-1'), 31.5 (C-8'), 28.7 (C-7'), 28.5 (C-6'), 22.5 (C-9'), 18.8 (C-2'), 18.7 (C-5'), 14.2 (N–CH_2_–CH_3_), 14.0 (C-10'). ESI-MS *m/z* (rel. int.): [M+H]^+^ 310 (100).

*1-(n-Propyl)-2-(3*′*-decynyl)-4(1H)-quinolone* (**8c**) was prepared from **3a** (1.0 g, 5.6 mmol) in THF (15 mL), LDA (3.1 mL, 5.6 mmol) and *N*-(*n*-propyl)isatoic anhydride (**7c**) (0.85 g, 4.2 mmol) in THF (10 mL) as a yellow oil (56%). IR (KBr, cm^−1^): 3420, 2929, 2856, 1627, 1600, 1489, 1468, 1428, 1177, 759. ^1^H-NMR *δ*: 8.45 (d, *J* = 8.0 Hz, 1H, H-5), 7.64 (t, *J* = 8.0 Hz, 1H, H-7), 7.46 (d, *J* = 8.4 Hz, 1H, H-8), 7.35 (t, *J* = 7.6 Hz, 1H, H-6), 6.29 (s, 1H, H-3), 4.13 (t, *J* = 8.0 Hz, 2H, N–CH_2_–CH_2_–CH_3_), 2.91 (t, *J* = 7.6 Hz, 2H, H-1'), 2.58 (t, *J* = 7.6 Hz, 2H, H-2'), 2.12 (t, *J* = 7.6 Hz, 2H, H-5'), 1.84 (m, 2H, N–CH_2_–CH_2_–CH_3_), 1.44 (quint, *J* = 7.6 Hz, 2H, H-6'), 1.35–1.23 (m, 6H, H-7'-9'), 1.08 (t, *J* = 7.6 Hz, 3H, N–CH_2_–CH_2_–CH_3_), 0.85 (t, *J* = 7.2 Hz, 3H, H-10'). ^13^C-NMR *δ*: 175.4 (C-4), 152.7 (C-2), 140.7 (C-8a), 132.1 (C-7), 126.8 (C-5), 126.6 (C-4a), 123.3 (C-6), 115.5 (C-8), 110.9 (C-3), 82.7 (C-4'), 77.1 (C-3'), 47.8 (N–CH_2_–CH_2_–CH_3_), 33.2 (C-1'), 31.4 (C-8'), 28.7 (C-7'), 28.5 (C-6'), 22.5 (C-9'), 22.1 (N–CH_2_–CH_2_–CH_3_), 18.8 (C-2'), 18.6 (C-5'), 14.0 (C-10'), 11.0 (N–CH_2_–CH_2_–CH_3_). ESI-MS *m/z* (rel. int.): [M+H]^+^ 324 (100).

*1-Methyl-2-(3'-undecynyl)-4(1H)-quinolone* (**8d**) was prepared from **3b** (1.0 g, 5.2 mmol) in THF (15 mL), LDA (2.9 mL, 5.2 mmol) and *N*-methylisatoic anhydride (**7a**) (0.69 g, 3.9 mmol) in THF (10 mL) as a yellow semi solid (58%). IR (KBr, cm^−1^): 3420, 2928, 2855, 1628, 1600, 1500, 1469, 1177, 759. ^1^H-NMR *δ*: 8.43 (d, *J* = 8.0 Hz, 1H, H-5), 7.65 (t, *J* = 8.0 Hz, 1H, H-7), 7.49 (d, *J* = 8.4 Hz, 1H, H-8), 7.36 (t, *J* = 7.6 Hz, 1H, H-6), 6.27 (s, 1H, H-3), 3.76 (s, 3H, N–CH_3_), 2.92 (t, *J* = 7.2 Hz, 2H, H-1'), 2.55 (t, *J* = 6.8 Hz, 2H, H-2'), 2.11 (t, *J* = 6.8 Hz, 2H, H-5'), 1.44 (quint, *J* = 7.2 Hz, 2H, H-6'), 1.33–1.21 (m, 8H, H-7'-10'), 0.86 (t, *J* = 6.8 Hz, 3H, H-11'). ^13^C-NMR *δ*: 177.4 (C-4), 153.1 (C-2), 141.8 (C-8a), 132.2 (C-7), 126.6 (C-5), 126.4 (C-4a), 123.5 (C-6), 115.4 (C-8), 111.1 (C-3), 82.8 (C-4'), 76.9 (C-3'), 34.4 (N–CH_3_), 34.0 (C-1'), 31.7 (C-9'), 29.0 (C-8'), 28.8 (C-7'), 28.7 (C-6'), 22.6 (C-10'), 18.6 (C-2'), 18.5 (C-5'), 14.1 (C-11'). ESI-MS *m/z* (rel. int.): [M+H]^+^ 310 (100).

*1-Ethyl-2-(3'-undecynyl)-4(1H)-quinolone* (**8e**) was prepared from **3b** (1.0 g, 5.2 mmol) in THF (15 mL), LDA (2.9 mL, 5.2 mmol) and *N*-ethylisatoic anhydride (**7b**) (0.74 g, 3.9 mmol) in THF (10 mL) as a yellow semi solid (62%). IR (KBr, cm^−1^): 3434, 2930, 2851, 1621, 1600, 1490, 1468, 1431, 1309, 759. ^1^H-NMR *δ*: 8.45 (d, *J* = 8.0 Hz, 1H, H-5), 7.65 (t, *J* = 8.0 Hz, 1H, H-7), 7.51 (d, *J* = 8.4 Hz, 1H, H-8), 7.35 (t, *J* = 7.2 Hz, 1H, H-6), 6.28 (s, 1H, H-3), 4.27 (q, *J* = 7.6 Hz, 2H, N–CH_2_–CH_3_), 2.91 (t, *J* = 7.2 Hz, 2H, H-1'), 2.59 (t, *J* = 6.8 Hz, 2H, H-2'), 2.12 (t, *J* = 6.8 Hz, 2H, H-5'), 1.44 (t, *J* = 7.2 Hz, 2H, N–CH_2_–CH_3_), 1.42 (quint, *J* = 6.8 Hz, 2H, H-6'), 1.31–1.21 (m, 8H, H-7'-10'), 0.85 (t, *J* = 6.8 Hz, 3H, H-11'). ^13^C-NMR *δ*: 177.1 (C-4), 151.9 (C-2), 141.1 (C-8a), 132.1 (C-7), 126.9 (C-5), 126.7 (C-4a), 123.2 (C-6), 115.4 (C-8), 110.8 (C-3), 82.4 (C-4'), 77.4 (C-3'), 41.3 (N–CH_2_–CH_3_), 33.0 (C-1'), 31.6 (C-9'), 29.0 (C-8'), 28.8 (C-7'), 28.6 (C-6'), 22.6 (C-10'), 18.8 (C-2'), 18.7 (C-5'), 14.2 (N–CH_2_–CH_3_), 14.0 (C-14'). ESI-MS *m/z* (rel. int.): [M+H]^+^ 324 (100).

*1-(n-Propyl)-2-(3'-undecynyl)-4(1H)-quinolone* (**8f**) was prepared from **3b** (1.0 g, 5.2 mmol) in THF (15 mL), LDA (2.9 mL, 5.2 mmol) and *N*-(*n*-propyl)isatoic anhydride (**7c**) (0.79 g, 3.9 mmol) in THF (10 mL) as a yellow oil (59%). IR (KBr, cm^−1^): 3426, 2929, 2856, 1627, 1600, 1488, 1468, 1427, 1177, 759. ^1^H-NMR *δ*: 8.46 (d, *J* = 8.0 Hz, 1H, H-5), 7.66 (t, *J* = 7.6 Hz, 1H, H-7), 7.48 (d, *J* = 8.0 Hz, 1H, H-8), 7.37 (t, *J* = 7.6 Hz, 1H, H-6), 6.36 (s, 1H, H-3), 4.13 (t, *J* = 8.0 Hz, 2H, N–CH_2_–CH_2_–CH_3_), 2.93 (t, *J* = 7.6 Hz, 2H, H-1'), 2.61 (t, *J* = 7.2 Hz, 2H, H-2'), 2.13 (t, *J* = 7.2 Hz, 2H, H-5'), 1.85 (sext, *J* = 8.0 Hz, 2H, N–CH_2_–CH_2_–CH_3_), 1.45 (quint, *J* = 7.2 Hz, 2H, H-6'), 1.33–1.21 (m, 8H, H-7'-10'), 1.08 (t, *J* = 7.2 Hz, 2H, N–CH_2_–CH_2_–CH_3_), 0.86 (t, *J* = 6.8 Hz, 3H, H-11'). ^13^C-NMR *δ*: 177.3 (C-4), 152.7 (C-2), 140.5 (C-8a), 132.3 (C-7), 126.7 (C-5), 126.5 (C-4a), 123.2 (C-6), 115.6 (C-8), 111.0 (C-3), 82.5 (C-4'), 77.0 (C-3'), 47.7 (N–CH_2_–CH_2_–CH_3_), 32.9 (C-1'), 31.5 (C-9'), 29.0 (C-8'), 28.7 (C-7'), 28.4 (C-6'), 22.6 (C-10'), 22.3 (N–CH_2_–CH_2_–CH_3_), 18.7 (C-2'), 18.6 (C-5'), 14.0 (C-11'), 11.1 (N–CH_2_–CH_2_–CH_3_). ESI-MS *m/z* (rel. int.): [M+H]^+^ 338 (100).

*1-Ethyl-2-(1'-decynyl)-4(1H)-quinolone* (**8g**) was prepared from **6a** (1.0 g, 5.6 mmol) in THF (15 mL), LDA (3.1 mL, 5.6 mmol) and *N*-ethylisatoic anhydride (**7b**) (0.80 g, 4.2 mmol) in THF (10 mL) as a light yellow semi solid (54%). IR (KBr, cm^−1^): 3372, 2928, 2855, 2234, 1625, 1598, 1488, 1421, 758. ^1^H-NMR *δ*: 8.45 (d, *J* = 8.0 Hz, 1H, H-5), 7.69 (t, *J* = 7.6 Hz, 1H, H-7), 7.50 (d, *J* = 8.0, Hz, 1H, H-8), 7.38 (t, *J* = 7.6 Hz, 1H, H-6), 6.58 (s, 1H, H-3), 4.53 (q, *J* = 7.2 Hz, 2H, N–CH_2_–CH_3_), 2.53 (t, *J* = 7.2 Hz, 2H, H-3'), 1.67 (quint, *J* = 7.2 Hz, 2H, H-4'), 1.49 (t, *J* = 7.2 Hz, 2H, N–CH_2_–CH_3_), 1.33–1.22 (m, 10H, H-5'-9'), 0.89 (t, *J* = 7.2 Hz, 3H, H-10'). ^13^C-NMR *δ*: 177.1 (C-4), 141.3 (C-8a), 137.5 (C-2), 132.5 (C-7), 126.8 (C-4a), 126.6 (C-5), 123.5 (C-6), 115.5 (C-8), 115.1 (C-3), 111.3 (C-2'), 74.5 (C-1'), 42.4 (N–CH_2_–CH_3_), 31.8 (C-8'), 29.2 (C-7'), 29.1 (C-6'), 29.0 (C-5'), 28.1 (C-4'), 22.6 (C-9'), 19.3 (C-3'), 14.2 (N–CH_2_–CH_3_), 14.0 (C-10'). ESI-MS *m/z* (rel. int.): [M+H]^+^ 310 (100).

*1-(n-Propyl)-2-(1'-decynyl)-4(1H)-quinolone* (**8h**) was prepared from **6a** (1.0 g, 5.6 mmol) in THF (15 mL), LDA (3.1 mL, 5.6 mmol) and *N*-(*n*-propyl)isatoic anhydride (**7c**) (0.85 g, 4.2 mmol) in THF (10 mL) as a light yellow semi solid (51%). IR (KBr, cm^−1^): 3430, 2927, 2855, 2235, 1624, 1598, 1488, 1420, 1177, 758. ^1^H-NMR *δ*: 8.45 (d, *J* = 8.0 Hz, 1H, H-5), 7.66 (t, *J* = 7.2 Hz, 1H, H-7), 7.44 (d, *J* = 8.0 Hz, 1H, H-8), 7.36 (t, *J* = 7.2 Hz, 1H, H-6), 6.56 (s, 1H, H-3), 4.40 (t, *J* = 7.6 Hz, 2H, N–CH_2_–CH_2_–CH_3_), 2.51 (t, *J* = 6.8 Hz, 2H, H-3'), 1.86 (m, 2H, N–CH_2_–CH_2_–CH_3_), 1.65 (quint, *J* = 6.8 Hz, 2H, H-4'), 1.33–1.21 (m, 10H, H-5'-9'), 1.03 (t, *J* = 7.2 Hz, 3H, N–CH_2_–CH_2_–CH_3_), 0.89 (t, *J* = 6.8 Hz, 3H, H-10'). ^13^C-NMR *δ*: 177.1 (C-4), 141.0 (C-8a), 138.2 (C-2),132.5 (C-7), 126.7 (C-4a), 126.5 (C-5), 123.7 (C-6), 115.4 (C-8), 115.2 (C-3), 111.0 (C-2'), 75.1 (C-1'), 47.9 (N–CH_2_–CH_2_–CH_3_), 31.7 (C-8'), 29.1 (C-7'), 29.0 (C-6'), 29.0 (C-5'), 28.1 (C-4'), 22.6 (C-9'), 22.3 (N–CH_2_–CH_2_–CH_3_), 19.8 (C-3'), 14.0 (C-10'), 11.0 (N–CH_2_–CH_2_–CH_3_). ESI-MS *m/z* (rel. int.): [M+H]^+^ 324 (100).

*1-(n-Butyl)-2-(1'-decynyl)-4(1H)-quinolone* (**8i**) was prepared from **6a** (1.0 g, 5.6 mmol) in THF (15 mL), LDA (3.1 mL, 5.6 mmol) and *N*-(*n*-butyl)isatoic anhydride (**7d**) (0.91 g, 4.2 mmol) in THF (10 mL) as a light yellow oil (54%). IR (KBr, cm^−1^): 3428, 2928, 2856, 2233, 1622, 1597, 1490, 1466, 1422, 759. ^1^H-NMR *δ*: 8.43 (d, *J* = 8.0 Hz, 1H, H-5), 7.66 (t, *J* = 7.6 Hz, 1H, H-7), 7.45 (d, *J* = 8.0, Hz, 1H, H-8), 7.33 (t, *J* = 7.6 Hz, 1H, H-6), 6.58 (s, 1H, H-3), 4.38 (t, *J* = 7.6 Hz, 2H, N–CH_2_–CH_2_–CH_2_–CH_3_), 2.53 (t, *J* = 6.8 Hz, 2H, H-3'), 1.78 (quint, *J* = 7.2 Hz, 2H, N–CH_2_–CH_2_–CH_2_–CH_3_), 1.65 (quint, *J* = 6.8 Hz, 2H, H-4'), 1.48 (m, 2H, N–CH_2_–CH_2_–CH_2_–CH_3_), 1.31–1.24 (m, 10H, H-5'-9'), 1.04 (t, *J* = 7.6 Hz, 3H, N–CH_2_–CH_2_–CH_2_–CH_3_), 0.87 (t, *J* = 6.8 Hz, 3H, H-10’). ^13^C-NMR *δ*: 177.1 (C-4), 141.3 (C-8a), 137.3 (C-2),132.5 (C-7), 126.7 (C-4a), 126.5 (C-5), 123.7 (C-6), 115.7 (C-8), 115.0 (C-3), 111.0 (C-2'), 74.4 (C-1'), 48.4 (N–CH_2_–CH_2_–CH_2_–CH_3_), 31.8 (C-8'), 31.4 (N–CH_2_–CH_2_–CH_2_–CH_3_), 29.1 (C-7'), 29.0 (C-6'), 29.0 (C-5'), 28.1 (C-4'), 22.6 (C-9'), 20.9 (N–CH_2_–CH_2_–CH_2_–CH_3_), 19.3 (C-3'), 14.3 (N–CH_2_–CH_2_–CH_2_–CH_3_), 14.0 (C-10'). ESI-MS *m/z* (rel. int.): [M+H]^+^ 338 (100).

*1-Methyl-2-(3'-dodecynyl)-4(1H)-quinolone* (**8j**) was prepared from **3c** (1.0 g, 4.8 mmol) in THF (15 mL), LDA (2.7 mL, 4.8 mmol) and *N*-methylisatoic anhydride (**7a**) (0.64 g, 3.6 mmol) in THF (10 mL) as a light yellow semi solid (60%). IR (KBr, cm^−1^): 3420, 2927, 2855, 1628, 1600, 1499, 1469, 759. ^1^H-NMR *δ*: 8.38 (dd, *J* = 8.0, 1.6 Hz, 1H, H-5), 7.61 (t, *J* = 8.4 Hz, 1H, H-7), 7.45 (d, *J* = 8.4 Hz, 1H, H-8), 7.32 (t, *J* = 7.6 Hz, 1H, H-6), 6.19 (s, 1H, H-3), 3.71 (s, 3H, N–CH_3_), 2.87 (t, *J* = 7.6 Hz, 2H, H-1'), 2.52 (t, *J* = 7.6 Hz, 2H, H-2'), 2.07 (t, *J* = 7.6 Hz, 2H, H-5'), 1.43 (quint, *J* = 7.2 Hz, 2H, H-6'), 1.31–1.20 (m, 10H, H-7'-11'), 0.84 (t, *J* = 6.8 Hz, 3H, H-12'). ^13^C-NMR *δ*: 177.6 (C-4), 152.9 (C-2), 141.8 (C-8a), 132.1 (C-7), 126.5 (C-5), 126.3 (C-4a), 123.3 (C-6), 115.4 (C-8), 111.1 (C-3), 82.6 (C-4'), 77.0 (C-3'), 34.3 (N–CH_3_), 33.9 (C-1'), 31.8 (C-10'), 29.1 (C-9'), 29.1 (C-8'), 29.0 (C-7'), 28.8 (C-6'), 22.6 (C-11'), 18.6 (C-2'), 18.4 (C-5'), 14.0 (C-12'). ESI-MS *m/z* (rel. int.): [M+H]^+^ 324 (100).

*1-Ethyl-2-(3'-dodecynyl)-4(1H)-quinolone* (**8k**) was prepared from **3c** (1.0 g, 4.8 mmol) in THF (15 mL), LDA (2.7 mL, 4.8 mmol) and *N*-ethylisatoic anhydride (**7b**) (0.69 g, 3.6 mmol) in THF (10 mL) as a white solid (55%). m.p. 66–68 °C. IR (KBr, cm^−1^): 3440, 2919, 2850, 1621, 1600, 1490, 1468, 1308, 769. ^1^H-NMR *δ*: 8.43 (d, *J* = 8.0 Hz, 1H, H-5), 7.64 (t, *J* = 8.0 Hz, 1H, H-7), 7.50 (d, *J* = 8.4 Hz, 1H, H-8), 7.34 (t, *J* = 7.2 Hz, 1H, H-6), 6.28 (s, 1H, H-3), 4.26 (q, *J* = 7.2 Hz, 2H, N–CH_2_–CH_3_), 2.90 (t, *J* = 7.6 Hz, 2H, H-1'), 2.57 (t, *J* = 7.2 Hz, 2H, H-2'), 2.10 (t, *J* = 6.8 Hz, 2H, H-5'), 1.43 (t, *J* = 7.6 Hz, N–CH_2_–CH_3_), 1.41 (quint, *J* = 7.2 Hz, 2H, H-6'), 1.31–1.20 (m, 10H, H-7'-11'), 0.85 (t, *J* = 6.8 Hz, 3H, H-12'). ^13^C-NMR *δ*: 177.5 (C-4), 152.8 (C-2), 140.5 (C-8a), 132.1 (C-7), 126.9 (C-5), 126.7 (C-4a), 123.2 (C-6), 115.4 (C-8), 110.9 (C-3), 82.7 (C-4'), 77.0 (C-3'), 41.2 (N–CH_2_–CH_3_), 33.0 (C-1'), 31.7 (C-10'), 29.1 (C-9'), 29.0 (C-8'), 28.8 (C-7'), 28.8 (C-6'), 22.5 (C-11'), 18.8 (C-2'), 18.6 (C-5'), 14.1 (N–CH_2_–CH_3_), 14.0 (C-12'). ESI-MS *m/z* (rel. int.): [M+H]^+^ 338 (100).

*1-(n-Propyl)-2-(3'-dodecynyl)-4(1H)-quinolone* (**8l**) was prepared from **3c** (1.0 g, 4.8 mmol) in THF (15 mL), LDA (2.7 mL, 4.8 mmol) and *N*-(*n*-propyl)isatoic anhydride (**7c**) (0.74 g, 3.6 mmol) in THF (10 mL) as a light yellow semi solid (51%). IR (KBr, cm^−1^): 3426, 2928, 2855, 1628, 1600, 1488, 1467, 1427, 759. ^1^H-NMR *δ*: 8.44 (d, *J* = 8.0 Hz, 1H, H-5), 7.66 (t, *J* = 8.0 Hz, 1H, H-7), 7.47 (d, *J* = 8.0 Hz, 1H, H-8), 7.36 (t, *J* = 7.6 Hz, 1H, H-6), 6.28 (s, 1H, H-3), 4.16 (t, *J* = 8.0 Hz, 2H, N–CH_2_–CH_2_–CH_3_), 2.90 (t, *J* = 7.6 Hz, 2H, H-1'), 2.59 (t, *J* = 7.2 Hz, 2H, H-2'), 2.13 (t, *J* = 7.6 Hz, 2H, H-5'), 1.85 (sext, *J* = 7.2 Hz, 2H, N–CH_2_–CH_2_–CH_3_), 1.43 (quint, *J* = 7.6 Hz, 2H, H-6'), 1.33–1.21 (m, 10H, H-7'-11'), 1.07 (t, *J* = 7.2 Hz, 3H, N–CH_2_–CH_2_–CH_3_), 0.86 (t, *J* = 6.8 Hz, 3H, H-12'). ^13^C-NMR *δ*: 175.8 (C-4), 152.5 (C-2), 140.9 (C-8a), 132.2 (C-7), 126.7 (C-5), 126.5 (C-4a), 123.1 (C-6), 115.4 (C-8), 110.8 (C-3), 82.6 (C-4'), 77.1 (C-3'), 47.8 (N–CH_2_–CH_2_–CH_3_), 33.1 (C-1'), 31.6 (C-10'), 29.1 (C-9'), 29.0 (C-8'), 29.0 (C-7'), 28.6 (C-6'), 22.6 (C-11'), 22.0 (N–CH_2_–CH_2_–CH_3_), 18.8 (C-2'), 18.6 (C-5'), 14.0 (C-14’), 10.9 (N–CH_2_–CH_2_–CH_3_). ESI-MS *m/z* (rel. int.): [M+H]^+^ 352 (100).

*1-(n-Butyl)-2-(3'-dodecynyl)-4(1H)-quinolone* (**8m**) was prepared from **3c** (1.0 g, 4.8 mmol) in THF (15 mL), LDA (2.7 mL, 4.8 mmol) and *N*-(*n*-butyl)isatoic anhydride (**7d**) (0.79 g, 3.6 mmol) in THF (10 mL) as a yellow oil (58%). IR (KBr, cm^−1^): 3425, 2927, 2855, 1628, 1600, 1488, 1467, 1427, 759. ^1^H-NMR *δ*: 8.44 (dd, *J* = 8.0, 1.6 Hz, 1H, H-5), 7.65 (td, *J* = 8.0, 1.6 Hz, 1H, H-7), 7.48 (d, *J* = 8.4 Hz, 1H, H-8), 7.35 (t, *J* = 7.6 Hz, 1H, H-6), 6.27 (s, 1H, H-3), 4.13 (t, *J* =8.0 Hz, 2H, N–CH_2_–(CH_2_)_2_–CH_3_), 2.91 (t, *J* = 7.2 Hz, 2H, H-1'), 2.58 (t, *J* = 7.2 Hz, 2H, H-2'), 2.11 (m, 2H, H-5'), 1.80 (quint, *J* = 7.2 Hz, 2H, N–CH_2_–CH_2_–CH_2_–CH_3_), 1.46 (m, 2H, N-(CH_2_)_2_–CH_2_–CH_3_), 1.41 (quint, *J* = 7.2 Hz, 2H, H-6'), 1.32–1.23 (m, 10H, H-7'-11'), 1.03 (t, *J* = 7.2 Hz, 3H, N-(CH_2_)_3_–CH_3_), 0.85 (t, *J* = 6.8 Hz, 3H, H-12'). ^13^C-NMR *δ*: 177.4 (C-4), 152.8 (C-2), 140.7 (C-8a), 132.1 (C-7), 126.8 (C-5), 126.6 (C-4a), 123.3 (C-6), 115.6 (C-8), 110.8 (C-3), 82.7 (C-4'), 77.1 (C-3'), 47.6 (N–CH_2_–(CH_2_)_2_–CH_3_), 33.2 (C-1'), 31.8 (C-10'), 30.8 (N–CH_2_–CH_2_–CH_2_–CH_3_), 29.1 (C-9'), 29.0 (C-8'), 28.8 (C-7'), 28.8 (C-6'), 22.6 (C-11'), 20.1 (N–(CH_2_)_2_–CH_2_–CH_3_), 18.8 (C-2'), 18.6 (C-5'), 14.1 (C-14’), 13.8 (N–(CH_2_)_3_–CH_3_). ESI-MS *m/z* (rel. int.): [M+H]^+^ 366 (100).

*1-Methyl-2-(1'-dodecynyl)-4(1H)-quinolone* (**8n**) was prepared from **6b** (1.5 g, 7.2 mmol) in THF (25 mL), LDA (4.0 mL, 7.2 mmol) and *N*-methylisatoic anhydride (**7a**) (0.96 g, 5.4 mmol) in THF (15 mL) as a light yellow semi-solid (51%). IR (KBr, cm^−1^): 3421, 2925, 2854, 2234, 1625, 1599, 1496, 1469, 757. ^1^H-NMR *δ*: 8.41 (d, *J* = 8.0 Hz, 1H, H-5), 7.66 (t, *J* = 7.6 Hz, 1H, H-7), 7.44 (d, *J* = 8.0, Hz, 1H, H-8), 7.36 (t, *J* = 7.6 Hz, 1H, H-6), 6.55 (s, 1H, H-3), 3.94 (s, 3H, N–CH_3_), 3.17 (t, *J* = 7.2 Hz, 2H, H-1'), 2.51 (t, *J* = 6.8 Hz, 2H, H-3'), 1.64 (quint, *J* = 6.8 Hz, 2H, H-4'), 1.46 (m, 2H, H-5'), 1.34–1.23 (m, 12H, H-6'-11'), 0.87 (t, *J* = 6.8 Hz, 3H, H-12'). ^13^C-NMR *δ*: 177.0 (C-4), 141.1 (C-8a), 137.4 (C-2), 132.4 (C-7), 126.8 (C-4a), 126.7 (C-5), 123.6 (C-6), 115.6 (C-8), 115.0 (C-3), 102.2 (C-2'), 74.6 (C-1'), 36.7 (N–CH_3_), 31.8 (C-10'), 29.5 (C-9'), 29.4 (C-8'), 29.2 (C-7'), 29.0 (C6'), 29.0 (C5'), 28.0 (C-4'), 22.6 (C-11'), 19.6 (C-3'), 14.0 (C-12'). ESI-MS *m/z* (rel. int.): [M+H]^+^ 324 (100).

*1-Methyl-2-(3'-tetradecynyl)-4(1H)-quinolone* (**8o**) was prepared from **3d** (1.0 g, 4.2 mmol) in THF (15 mL), LDA (2.4 mL, 4.2 mmol) and *N*-methylisatoic anhydride (**7a**) (0.57 g, 3.2 mmol) in THF (10 mL) as a light yellow solid (60%). m.p. 43–45 °C. IR (KBr, cm^−1^): 3422, 2922, 2854, 1632, 1598, 1469, 1444, 760. ^1^H-NMR *δ*: 8.39 (d, *J* = 8.0 Hz, 1H, H-5), 7.62 (t, *J* = 8.0 Hz, 1H, H-7), 7.46 (d, *J* = 8.4 Hz, 1H, H-8), 7.33 (t, *J* = 7.2 Hz, 1H, H-6), 6.21 (s, 1H, H-3), 3.72 (s, 3H, N–CH_3_), 2.88 (t, *J* = 7.2 Hz, 2H, H-1'), 2.50 (t, *J* = 7.6 Hz, 2H, H-2’), 2.10 (t, *J* = 7.2 Hz, 2H, H-5'), 1.41 (quint, *J* = 6.8 Hz, 2H, H-6'), 1.34–1.21 (m, 14H, H-7'-13'), 0.85 (t, *J* = 6.8 Hz, 3H, H-14'). ^13^C-NMR *δ*: 177.6 (C-4), 152.9 (C-2), 141.8 (C-8a), 132.1 (C-7), 126.5 (C-5), 126.3 (C-4a), 123.3 (C-6), 115.4 (C-8), 111.1 (C-3), 82.7 (C-4'), 77.1 (C-3'), 34.3 (N–CH_3_), 33.9 (C-1'), 31.8 (C-12'), 29.2 (C-11'), 29.1 (C-10'), 29.1 (C-9'), 29.0 (C-8'), 29.0 (C-7'), 28.8 (C-6'), 22.6 (C-13'), 18.6 (C-2'), 18.4 (C-5'), 14.0 (C-14'). ESI-MS *m/z* (rel. int.): [M+H]^+^ 352 (100).

*1-Ethyl-2-(3'-tetradecynyl)-4(1H)-quinolone* (**8p**) was prepared from **3d** (1.0 g, 4.2 mmol) in THF (15 mL), LDA (2.4 mL, 4.2 mmol) and *N*-ethylisatoic anhydride (**7b**) (0.61 g, 3.2 mmol) in THF (10 mL) as white needles (56%). m.p. 74–76 °C. IR (KBr, cm^−1^): 3424, 2917, 2850, 1621, 1600, 1468, 1430, 1308, 760. ^1^H-NMR *δ*: 8.44 (d, *J* = 8.0 Hz, 1H, H-5), 7.63 (t, *J* = 8.0 Hz, 1H, H-7), 7.50 (d, *J* = 8.4 Hz, 1H, H-8), 7.35 (t, *J* = 7.2 Hz, 1H, H-6), 6.29 (s, 1H, H-3), 4.24 (q, *J* = 7.2 Hz, 2H, N–CH_2_–CH_3_), 2.91 (t, *J* = 7.2 Hz, 2H, H-1'), 2.58 (t, *J* = 7.2 Hz, 2H, H-2'), 2.09 (t, *J* = 6.4 Hz, 2H, H-5'), 1.42 (t, *J* = 7.2 Hz, N–CH_2_–CH_3_), 1.40 (quint, *J* = 7.2 Hz, 2H, H-6'), 1.31–1.21 (m, 14H, H-7'-13'), 0.85 (t, *J* = 6.8 Hz, 3H, H-14'). ^13^C-NMR *δ*: 177.5 (C-4), 152.7 (C-2), 140.8 (C-8a), 132.1 (C-7), 126.6 (C-5), 126.4 (C-4a), 123.3 (C-6), 115.4 (C-8), 111.1 (C-3), 82.7 (C-4'), 77.0 (C-3'), 41.3 (N–CH_2_–CH_3_), 33.1 (C-1'), 31.8 (C-12'), 29.2 (C-11'), 29.2 (C-10'), 29.1 (C-9'), 29.0 (C-8'), 29.0 (C-7'), 28.8 (C-6'), 22.6 (C-13'), 18.8 (C-2'), 18.7 (C-5'), 14.2 (N–CH_2_–CH_3_), 14.0 (C-14'). ESI-MS *m/z* (rel. int.): [M+H]^+^ 366 (100).

*1-(n-Propyl)-2-(3'-tetradecynyl)-4(1H)-quinolone* (**8q**) was prepared from **3d** (1.0 g, 4.2 mmol) in THF (15 mL), LDA (2.4 mL, 4.2 mmol) and *N*-(*n*-propyl)isatoic anhydride (**7c**) (0.66 g, 3.2 mmol) in THF (10 mL) as a light yellow semi-solid (55%). IR (KBr, cm^−1^): 3430, 2926, 2854, 1629, 1600, 1488, 1467, 1227, 759. ^1^H-NMR *δ*: 8.48 (d, *J* = 8.0 Hz, 1H, H-5), 7.67 (t, *J* = 7.6 Hz, 1H, H-7), 7.49 (d, *J* = 8.4 Hz, 1H, H-8), 7.38 (t, *J* = 7.6 Hz, 1H, H-6), 6.43 (s, 1H, H-3), 4.17 (t, *J* = 8.0 Hz, 2H, N–CH_2_–CH_2_–CH_3_), 2.94 (t, *J* = 7.6 Hz, 2H, H-1'), 2.59 (t, *J* = 7.2 Hz, 2H, H-2'), 2.11 (t, *J* = 7.2 Hz, 2H, H-5'), 1.86 (sext, *J* = 7.6 Hz, 2H, N–CH_2_–CH_2_–CH_3_), 1.43 (quint, *J* = 7.2 Hz, 2H, H-6'), 1.33–1.22 (m, 14H, H-7'-13'), 1.09 (t, *J* = 7.2 Hz, 2H, N–CH_2_–CH_2_–CH_3_), 0.88 (t, *J* = 6.8 Hz, 3H, H-14'). ^13^C-NMR *δ*: 175.4 (C-4), 152.5 (C-2), 141.0 (C-8a), 132.1 (C-7), 126.7 (C-5), 126.5 (C-4a), 123.3 (C-6), 115.3 (C-8), 111.0 (C-3), 82.6 (C-4'), 77.0 (C-3’), 47.6 (N–CH_2_–CH_2_–CH_3_), 33.1 (C-1'), 31.5 (C-12'), 29.3 (C-11'), 29.2 (C-10'), 29.2 (C-9'), 29.1 (C-8'), 29.0 (C-7'), 28.4 (C-6'), 22.6 (C-13'), 22.1 (N–CH_2_–CH_2_–CH_3_), 18.8 (C-2'), 18.6 (C-5'), 14.0 (C-14'), 11.0 (N–CH_2_–CH_2_–CH_3_). ESI-MS *m/z* (rel. int.): [M+H]^+^ 380 (100).

*1-Methyl-2-(1*′*-tetradecynyl)-4(1H)-quinolone* (**8r**) was prepared from **6c** (1.5 g, 6.4 mmol) in THF (25 mL), LDA (3.5 mL, 6.4 mmol) and *N*-methylisatoic anhydride (**7a**) (0.85 g, 4.8 mmol) in THF (15 mL) as a white solid (57%). M.p. 41–43 °C. IR (KBr, cm^−1^): 3405, 2923, 2849, 2233, 1622, 1595, 1496, 1468, 777. ^1^H-NMR *δ*: 8.38 (dd, *J* = 8.0, 1.6 Hz, 1H, H-5), 7.63 (td, *J* = 8.0, 1.6 Hz, 1H, H-7), 7.42 (d, *J* = 8.0 Hz, 1H, H-8), 7.33 (t, *J* = 7.6 Hz, 1H, H-6), 6.51 (s, 1H, H-3), 3.92 (s, 3H, N–CH_3_), 2.49 (t, *J* = 7.2 Hz, 2H, H-3'), 1.63 (quint, *J* = 7.2 Hz, 2H, H-4'), 1.44 (m, 2H, H-5'), 1.34–1.21 (m, 16H, H-6'-13'), 0.85 (t, *J* = 6.8 Hz, 3H, H-14'). ^13^C-NMR *δ*: 177.1 (C-4), 141.1 (C-8a), 137.3 (C-2), 132.3 (C-7), 126.8 (C-4a), 126.6 (C-5), 123.6 (C-6), 115.6 (C-8), 115.0 (C-3), 102.0 (C-2'), 74.8 (C-1'), 36.7 (N–CH_3_), 31.8 (C-12'), 29.6 (C-11'), 29.6 (C-10'), 29.5 (C-9'), 29.4 (C-8'), 29.2 (C-7'), 29.0 (C6'), 28.9 (C5'), 27.9 (C-4'), 22.6 (C-13'), 19.6 (C-3'), 14.0 (C-14'). ESI-MS *m/z* (rel. int.): [M+H]^+^ 352 (100).

*1-Methyl-2-(3*′*-hexadecynyl)-4(1H)-quinolone* (**8s**) was prepared from **3e** (1.5 g, 5.7 mmol) in THF (25 mL), LDA (3.2 mL, 5.7 mmol) and *N*-methylisatoic anhydride (**7a**) (0.76 g, 4.3 mmol) in THF (15 mL) as a light yellow solid (59%). M.p. 58–61 °C. IR (KBr, cm^−1^): 3432, 2922, 2853, 1631, 1597, 1469, 1444, 761. ^1^H-NMR *δ*: 8.46 (d, *J* = 8.0 Hz, 1H, H-5), 7.73 (t, *J* = 7.6 Hz, 1H, H-7), 7.58 (d, *J* = 8.0 Hz, 1H, H-8), 7.44 (t, *J* = 7.6 Hz, 1H, H-6), 6.56 (s, 1H, H-3), 3.85 (s, 3H, N–CH_3_), 3.00 (t, *J* = 7.6 Hz, 2H, H-1'), 2.60 (t, *J* = 7.6 Hz, 2H, H-2’), 2.12 (t, *J* = 7.2 Hz, 2H, H-5'), 1.41 (m, 2H, H-6'), 1.32–1.19 (m, 18H, H-7'-15'), 0.88 (t, *J* = 6.4 Hz, 3H, H-16'). ^13^C-NMR *δ*: 175.6 (C-4), 155.3 (C-2), 141.7 (C-8a), 133.3 (C-7), 126.4 (C-5), 126.2 (C-4a), 124.4 (C-6), 115.9 (C-8), 110.5 (C-3), 83.4 (C-4'), 76.5 (C-3'), 35.6 (N–CH_3_), 34.1 (C-1'), 31.9 (C-14'), 29.7 (C-13'), 29.6 (C-12'), 29.6 (C-11'), 29.5 (C-10'), 29.3 (C-9'), 29.1 (C-8'), 28.9 (C-7'), 28.9 (C-6'), 22.7 (C-15'), 18.8 (C-2'), 18.6 (C-5'), 14.1 (C-16'). ESI-MS *m/z* (rel. int.): [M+H]^+^ 380 (100).

*1-Methyl-2-(1'-pentadecynyl)-4(1H)-quinolone* (**8t**) was prepared from **6d** (1.5 g, 6.0 mmol) in THF (25 mL), LDA (3.3 mL, 6.0 mmol) and *N*-methylisatoic anhydride (**7a**) (0.80 g, 4.5 mmol) in THF (15 mL) as white needles (48%). M.p. 67–69 °C. IR (KBr, cm^−1^): 3401, 2915, 2851, 2239, 1618, 1596, 1472, 748. ^1^H-NMR *δ*: 8.42 (d, *J* = 8.4, Hz, 1H, H-5), 7.69 (td, *J* = 8.0, 1.6 Hz, 1H, H-7), 7.48 (d, *J* = 8.0 Hz, 1H, H-8), 7.35 (t, *J* = 7.6 Hz, 1H, H-6), 6.55 (s, 1H, H-3), 3.96 (s, 3H, N–CH_3_), 2.52 (t, *J* = 7.2 Hz, 2H, H-3'), 1.66 (quint, *J* = 7.6 Hz, 2H, H-4'), 1.46 (quint, *J* = 7.2 Hz, 2H, H-5'), 1.35–1.21 (m, 18H, H-6'-14'), 0.87 (t, *J* = 6.8 Hz, 3H, H-15'). ^13^C-NMR *δ*: 177.2 (C-4), 141.0 (C-8a), 136.6 (C-2), 132.2 (C-7), 126.7 (C-4a), 126.6 (C-5), 123.7 (C-6), 115.7 (C-8), 115.1 (C-3), 102.1 (C-2'), 74.9 (C-1'), 36.5 (N–CH_3_), 31.8 (C-13'), 29.6 (C-12'), 29.5 (C-11'), 29.5 (C-10'), 29.3 (C-9'), 29.3 (C-8'), 29.1 (C-7'), 29.1 (C6'), 28.9 (C5'), 28.0 (C-4'), 22.6 (C-14'), 19.5 (C-3'), 14.1 (C-14'). ESI-MS *m/z* (rel. int.): [M+H]^+^ 366 (100).

*1-Cyclopropyl-2-[(E)-12-bromodec-1'-enyl]-4(1H)-quinolone* (**18a**) was prepared from **17a** (1.0 g, 3.5 mmol) in THF (15 mL), LDA (1.9 mL, 3.5 mmol) and *N*-cyclopropylisatoic anhydride (**12**) (0.52 g, 2.6 mmol) in THF (10 mL) as a light yellow solid (49%). M.p. 85–87 °C. IR (KBr, cm^−1^): 3425, 2926, 2850, 1648, 1616, 1594, 1475, 1416, 1310, 1137, 1034, 966, 888, 760. ^1^H-NMR *δ*: 8.39 (dd, *J* = 8.0, 1.6 Hz, 1H, H-5), 7.90 (d, *J* = 8.4 Hz, 1H, H-8), 7.64 (td, *J* = 8.0, 1.6 Hz, 1H, H-7), 7.35 (t, *J* = 7.6 Hz, 1H, H-6), 6.67 (d, *J* = 16.0 Hz, 1H, H-1'), 6.55 (s, 1H, H-3), 6.45 (dt, *J* = 16.0, 6.8 Hz, 1H, H-2'), 3.41 (t, *J* = 6.8 Hz, 2H, H-12'), 3.28 (sept, *J* = 4.0 Hz, 1H, N–CH–(CH_2_)_2_), 2.30 (q, *J* = 6.8 Hz, 2H, H-3'), 1.83 (quint, *J* = 6.8 Hz, 2H, H-11'), 1.51 (quint, *J* = 6.8 Hz, 2H, H-4'), 1.41–1.23 (m, 12H, H-5'-10'), 0.90 (m, 4H, N–CH–(CH_2_)_2_). ^13^C-NMR *δ*: 178.2 (C-4), 153.0 (C-2), 142.2 (C-2'), 139.6 (C-8a), 131.5 (C-7), 126.4 (C-4a), 126.2 (C-5), 124.9 (C-1'), 123.4 (C-6), 117.4 (C-8), 108.2 (C-3), 34.1 (C-12'), 33.1 (C-3'), 32.7 (C-11'), 29.9 (C-8'), 29.4 (C-7'), 29.4 (C-9'), 29.3 (C-6'), 29.1 (C-5'), 28.6 (C-4'), 28.1 (C-10'), 24.7 (N–CH–(CH_2_)_2_), 2×12.4 (N–CH–(CH_2_)_2_). ESI-MS *m/z* (rel. int.): [M+H+2]^+^ 432 (100), [M+H]^+^ 430 (94). 

*1-Cyclopropyl-2-[(E)-13-bromotridec-1'-enyl)-4(1H)-quinolone* (**18b**) was prepared from **17b** (1.0 g, 3.3 mmol) in THF (15 mL), LDA (1.8 mL, 3.3 mmol) and *N*-cyclopropylisatoic anhydride (**12**) (0.5 g, 2.5 mmol) in THF (10 mL) as a light yellow solid (55%). M.p. 61–63 °C. IR (KBr, cm^−1^): 3422, 3020, 2918, 2851, 1653, 1630, 1596, 1571, 1479, 1463, 1413, 1135, 1034, 973, 760. ^1^H-NMR *δ*: 8.38 (dd, *J* = 8.0, 1.6 Hz, 1H, H-5), 7.89 (d, *J* = 8.4 Hz, 1H, H-8), 7.64 (td, *J* = 8.0, 1.6 Hz, 1H, H-7), 7.35 (t, *J* = 7.6 Hz, 1H, H-6), 6.66 (d, *J* = 16.0 Hz, 1H, H-1'), 6.50 (s, 1H, H-3), 6.44 (dt, *J* = 16.0, 6.8 Hz, 1H, H-2'), 3.41 (t, *J* = 6.8 Hz, 2H, H-13'), 3.27 (sept, *J* = 4.0 Hz, 1H, N–CH–(CH_2_)_2_), 2.30 (q, *J* = 6.8 Hz, 2H, H-3'), 1.85 (quint, *J* = 6.8 Hz, 2H, H-12'), 1.51 (quint, *J* = 6.8 Hz, 2H, H-4'), 1.43–1.23 (m, 14H, H-5'-11'), 0.90 (m, 4H, N–CH–(CH_2_)_2_). ^13^C-NMR *δ*: 178.2 (C-4), 152.9 (C-2), 142.1 (C-2'), 139.5 (C-8a), 131.4 (C-7), 126.4 (C-4a), 126.2 (C-5), 124.9 (C-1'), 123.3 (C-6), 117.4 (C-8), 108.2 (C-3), 34.1 (C-13'), 33.1 (C-3'), 32.7 (C-12'), 29.5 (C-9'), 29.5 (C-8'), 29.3 (C-10'), 29.3 (C-7'), 29.2 (C-6'), 28.7 (C-5'), 28.6 (C-4'), 28.1 (C-11'), 24.9 (N–CH–(CH_2_)_2_), 2 × 12.4 (N–CH–(CH_2_)_2_). ESI-MS *m/z* (rel. int.): [M+H+2]^+^ 446 (100), [M+H]^+^ 444 (94).

*1-Methyl-2-[(E)-12-bromodec-1'-enyl]-4(1H)-quinolone* (**19a**) was prepared from **17a** (1.5 g, 5.2 mmol) in THF (25 mL), LDA (2.9 mL, 5.2 mmol) and *N*-methylisatoic anhydride (**7a**) (0.69 g, 3.9 mmol) in THF (10 mL) as a yellow semi solid (63%). IR (KBr, cm^−1^): 3422, 2925, 2852, 1620, 1597, 1467, 1438, 761. ^1^H-NMR *δ*: 8.44 (d, *J* = 8.0 Hz, 1H, H-5), 7.67 (t, *J* = 7.2 Hz, 1H, H-7), 7.49 (t, *J* = 8.4 Hz, 1H, H-8), 7.38 (t, *J* = 7.2 Hz, 1H, H-6), 6.46 (s, 1H, H-3), 6.43 (d, *J* = 16.0 Hz, 1H, H-1'), 6.37 (dt, *J* = 16.0, 6.4 Hz, 1H, H-2'), 3.76 (s, 3H, N–CH_3_), 3.41 (t, *J* = 6.4 Hz, 2H, H-12'), 2.28 (q, *J* = 6.8 Hz, 2H, H-3'), 1.85 (quint, *J* = 6.8 Hz, 2H, H-11'), 1.50 (quint, *J* = 6.8 Hz, 2H, H-4'), 1.41 (quint, *J* = 6.8 Hz, 2H, H-10'), 1.37–1.23 (m, 10H, H-5'-9'). ^13^C-NMR *δ*: 177.9 (C-4), 152.5 (C-2), 142.0 (C-2'), 141.4 (C-8a), 132.2 (C-7), 126.6 (C-4a), 126.5 (C-5), 123.8 (C-1'), 123.5 (C-6), 115.5 (C-8), 109.4 (C-3), 35.5 (N–CH_3_), 34.1 (C-12'), 33.1 (C-3'), 32.7 (C-11'), 29.4 (C-8'), 29.3 (C-9'), 29.3 (C-7'), 29.1 (C-6'), 28.7 (C-5'), 28.5 (C-4'), 28.1 (C-10'). ESI-MS *m/z* (rel. int.): [M+H]^+^ 404 (100), [M+H+2]^+^ 406 (92).

*1-Methyl-2-[(E)-13-bromotridec-1*'*-enyl)-4(1H)-quinolone* (**19b**) was prepared from **17b** (1.0 g, 3.3 mmol) in THF (15 mL), LDA (1.8 mL, 3.3 mmol) and *N*-methylisatoic anhydride (**7a**) (0.44 g, 2.5 mmol) in THF (10 mL) as a yellow semi solid (47%). IR (KBr, cm^−1^): 3420, 2925, 2852, 1622, 1597, 1496, 1468, 760. ^1^H-NMR *δ*: 8.45 (d, *J* = 8.0 Hz, 1H, H-5), 7.70 (t, *J* = 7.6 Hz, 1H, H-7), 7.52 (t, *J* = 8.0 Hz, 1H, H-8), 7.40 (t, *J* = 7.6 Hz, 1H, H-6), 6.48 (s, 1H, H-3), 6.44 (d, *J* = 16.0 Hz, 1H, H-1'), 6.37 (dt, *J* = 16.0, 6.4 Hz, 1H, H-2'), 3.79 (s, 3H, N–CH_3_), 3.41 (t, *J* = 6.8 Hz, 2H, H-13'), 2.29 (q, *J* = 7.2 Hz, 2H, H-3'), 1.86 (quint, *J* = 6.8 Hz, 2H, H-12'), 1.52 (quint, *J* = 6.8 Hz, 2H, H-4'), 1.43 (quint, *J* = 6.8 Hz, 2H, H-11'), 1.37–1.22 (m, 12H, H-5'-10'). ^13^C-NMR *δ*: 177.4 (C-4), 152.3 (C-2), 142.4 (C-2'), 141.4 (C-8a), 132.4 (C-7), 126.7 (C-4a), 126.6 (C-5), 123.9 (C-1'), 123.6 (C-6), 115.5 (C-8), 109.4 (C-3), 35.6 (N–CH_3_), 34.1 (C-13'), 33.2 (C-3'), 32.8 (C-12'), 29.5 (C-9'), 29.5 (C-8'), 29.3 (C-10'), 29.3 (C-7'), 29.1 (C-6'), 28.7 (C-5'), 28.6 (C-4'), 28.1 (C-11'). ESI-MS *m/z* (rel. int.): [M+H]^+^ 418 (100), [M+H+2]^+^ 420 (94).

*1-Cyclopropyl-2-[(E)-1*′*-tridecenyl]-4(1H)-quinolone* (**22a**) was prepared from **21a** (1.5 g, 6.7 mmol) in THF (25 mL), LDA (3.7 mL, 6.7 mmol) and *N*-cyclopropylisatoic anhydride (**12**) (1.0 g, 5.0 mmol) in THF (20 mL) as white needles (56%). M.p. 86–88 °C. IR (KBr, cm^−1^): 3425, 2922, 2851, 1617, 1595, 1571, 1479, 1465, 1420, 1310, 1136, 1031, 966, 759. ^1^H-NMR *δ*: 8.37 (dd, *J* = 8.0, 1.6 Hz, 1H, H-5), 7.88 (d, *J* = 8.4 Hz, 1H, H-8), 7.62 (td, *J* = 8.0, 1.6 Hz, 1H, H-7), 7.33 (t, *J* = 8.0 Hz, 1H, H-6), 6.65 (d, *J* = 16.0 Hz, 1H, H-1'), 6.48 (s, 1H, H-3), 6.42 (dt, *J* = 16.0, 6.8 Hz, 1H, H-2′), 3.26 (sept, *J* = 4.0 Hz, 1H, N–CH–(CH_2_)_2_), 2.29 (q, *J* = 6.8 Hz, 2H, H-3′), 1.52 (quint, *J* = 6.8 Hz, 2H, H-4'), 1.36–1.24 (m, 16H, H-5'-12'), 0.86–0.90 (m, 7H, H-13', N–CH–(CH_2_)_2_). ^13^C-NMR *δ*: 178.2 (C-4), 152.8 (C-2), 142.1 (C-8a), 139.3 (C-2'), 131.4 (C-7), 126.5 (C-4a), 126.1 (C-5), 124.9 (C-1'), 123.3 (C-6), 117.4 (C-8), 108.2 (C-3), 33.1 (C-3'), 31.8 (C-11'), 29.8 (N–CH–(CH_2_)_2_), 29.6 (C-10'), 29.5 (C-9), 29.5 (C-8'), 29.4 (C-7'), 29.3 (C6'), 29.2 (C5'), 28.6 (C-4'), 22.6 (C-12'), 14.0 (C-13'), 12.3 (N–CH–(CH_2_)_2_). ESI-MS *m/z* (rel. int.): [M+H]^+^ 366 (100).

*1-Cyclopropyl-2-[(E)-1′-tetradecenyl]-4(1H)-quinolone* (**22b**) was prepared from **21b** (1.5 g, 6.3 mmol) in THF (25 mL), LDA (3.5 mL, 6.3 mmol) and *N*-cyclopropylisatoic anhydride (**12**) (0.96 g, 4.7 mmol) in THF (20 mL) as white crystals (50%). M.p. 96–98 °C. IR (KBr, cm^−1^): 3422, 2919, 2848, 1620, 1598, 1572, 1482, 1465, 1419, 1306, 1132, 1037, 966, 750. ^1^H-NMR *δ*: 8.36 (d, *J* = 8.0 Hz, 1H, H-5), 7.87 (d, *J* = 8.4 Hz, 1H, H-8), 7.62 (td, *J* = 8.0, 1.6 Hz, 1H, H-7), 7.32 (t, *J* = 8.0 Hz, 1H, H-6), 6.65 (d, *J* = 16.0 Hz, 1H, H-1'), 6.46 (s, 1H, H-3), 6.42 (dt, *J* = 16.0, 6.8 Hz, 1H, H-2'), 3.25 (sept, *J* = 4.0 Hz, 1H, N–CH–(CH_2_)_2_), 2.28 (q, *J* = 6.8 Hz, 2H, H-3'), 1.51 (quint, *J* = 6.8 Hz, 2H, H-4'), 1.37–1.21 (m, 18H, H-5'-13'), 0.86–0.91 (m, 7H, H-14', N–CH–(CH_2_)_2_). ^13^C-NMR *δ*: 178.2 (C-4), 152.8 (C-2), 142.1 (C-8a), 139.3 (C-2'), 131.3 (C-7), 126.5 (C-4a), 126.1 (C-5), 124.9 (C-1'), 123.2 (C-6), 117.4 (C-8), 108.3 (C-3), 33.1 (C-3'), 31.8 (C-12'), 29.8 (N–CH–(CH_2_)_2_), 29.6 (C-11'), 29.6 (C-10'), 29.6 (C-9), 29.5 (C-8'), 29.4 (C-7'), 29.3 (C6'), 29.2 (C5'), 28.7 (C-4'), 22.6 (C-13'), 14.1 (C-14'), 12.3 (N–CH–(CH_2_)_2_). ESI-MS *m/z* (rel. int.): [M+H]^+^ 380 (100). 

*1-Cyclopropyl-2-(3*′*-undecynyl)-4(1H)-quinolone* (**23**) was prepared from **3b** (1.5 g, 7.7 mmol) in THF (25 mL), LDA (4.3 mL, 7.7 mmol) and *N*-cyclopropylisatoic anhydride (**12**) (1.17 g, 5.8 mmol) in THF (25 mL) as a yellow oil (48%). IR (KBr, cm^−1^): 3424, 2928, 2855, 1628, 1601, 1553, 1481, 1466, 1420, 1311, 1132, 1044, 759. ^1^H-NMR *δ*: 8.34 (dd, *J* = 8.0, 1.6 Hz, 1H, H-5), 7.87 (d, *J* = 8.4 Hz, 1H, H-8), 7.60 (td, *J* = 8.0, 1.6 Hz, 1H, H-7), 7.31 (t, *J* = 6.8 Hz, 1H, H-6), 6.25 (s, 1H, H-3), 3.27 (sept, *J* = 4.0 Hz, 1H, N–CH–(CH_2_)_2_), 3.17 (t, *J* = 7.2 Hz, 2H, H-1'), 2.54 (m, 2H, H-2'), 2.07 (m, 2H, H-5'), 1.41 (quint, *J* = 6.8 Hz, 2H, H-6'), 1.26–1.15 (m, 6H, H-7'-9'), 0.93 (m, 4H, N–CH–(CH_2_)_2_), 0.85 (t, *J* = 6.8 Hz, 3H, H-10'). ^13^C-NMR *δ*: 178.1 (C-4), 155.6 (C-2), 142.8 (C-8a), 131.1 (C-7), 126.3 (C-4a), 126.1 (C-5), 123.1 (C-6), 117.6 (C-8), 111.0 (C-3), 82.9 (C-4'), 77.5 (C-3'), 33.0 (C-1'), 31.6 (C-8'), 29.1 (C-7'), 28.7 (C-6'), 28.7 (C-5'), 26.8 (N–CH–(CH_2_)_2_), 22.5 (C-9'), 18.6 (C-2'), 14.0 (C-10'), 12.3 (N–CH–(CH_2_)_2_). ESI-MS *m/z* (rel. int.): [M+H]^+^ 336 (100).

### 3.3. Biological Evaluation

#### 3.3.1. *In Vitro* Antibacterial Activities against Fast Growing Strains of Mycobacteria and EMRSA-15 and EMRSA-16

*In vitro* *M. smegmatis*, *M. fortuitum*, *M. phlei*, EMRSA-15 and -16 inhibitory effect of the 4(1*H*)-quinolone derivatives was assessed in 96-well plates using the broth dilution assay according to the previously reported protocols [7,23]. The plates containing test compounds, the antibiotic drugs and growth media were incubated at 37 °C for 72 h for the rapidly-growing mycobacterial strains and 18 h for EMRSA-15 and -16. A methanolic solution of MTT (0.05%) was used to determine the MIC by a colour change from yellow to blue. Tests were carried out in triplicate and all MIC values were determined in separate duplicate experiments. Tetracycline and norfloxacin were used as a positive control for the EMRSA strains and isoniazid and ethambutol for the mycobacterial strains.

#### 3.3.2. Spot-Culture Growth Inhibition Assay (SPOTi) against *M. bovis* BCG

A diluted culture (~500 viable cells) of *M. bovis* BCG was spotted into 10% OADC supplemented Middlebrook 7H10 agar medium in a 24-well plate having various concentrations of the 4(1*H*)-quinolone derivatives and isoniazid. The plates were incubated at 37 °C for two weeks and the MIC values were determined visually as the minimum concentrations where no growth was observed [24].

#### 3.3.3. *M. tuberculosis* MurE Inhibition Assay

The *M. tuberculosis* MurE ligase activity was determined by the phosphate colorimetric detection method as previously reported [7]. The compounds were tested at concentrations of 1000, 300, 100 and 30 µM and the amount of phosphate released was determined by means of a phosphate calibration curve using the Pi ColorLock kit reagents (Innova Biosciences, Cambridge, UK). Isoniazid was used as a negative control and IC_50_ values were determined by extrapolation from the plot of percent inhibition *vs.* concentration.

#### 3.3.4. Cytotoxicity Assay

The cytotoxic activity of the synthetic compounds was determined by means of an XTT Cell Proliferation Kit II (Roche Diagnostics, Mannheim, Germany) using the human diploid embryonic lung cell line MRC-5 [11]. The quinolone derivatives were tested at concentrations of 100, 60, 30 and 10 µM in triplicate and the percentage of viable cells at each concentration was determined by comparing with the control.

## 4. Conclusions

Using the SAR information gained from our previous studies, a further group of quinolone derivatives possessing a diverse set of alkynyl/(*E*)-alkenyl substituents at C-2 of the quinolone nucleus were synthesized. Although the antimycobacterial potencies are comparable to those of the previously reported alkenyl analoges, the new series of alkynyl bearing compounds displayed higher selectivity towards mycobacteria and MRSA strains compared to MRC-5 cells. It is reasonable to conclude that the improved cytotoxicity of this group of analoges is due to the introduction of a triple bond in the aliphatic side chain of the quinolone nucleus. The selectivity shown by this class of new quinolones make them promising leads for synthesizing further compounds with improved antibacterial activity. Compounds with a terminal bromine atom at the side chain of *N*-alkyl-2-alkynyl/(*E*)-alkenyl-4-(1*H*)-quinolones with improved antimycobacterial activity will be explored due their increased probability of covalent bonding to particular target proteins.
